# A Review on Sulfonamide Complexes with Metals: Their Pharmacological Potential as Anticancer Drugs

**DOI:** 10.3390/ph18091414

**Published:** 2025-09-19

**Authors:** Przemysław Rozbicki, Danuta Branowska

**Affiliations:** Institute of Chemical Sciences, Faculty of Science, University of Siedlce, 3 Maja 54 Street, PL-08-1103 Siedlce, Poland

**Keywords:** sulfonamide, complex with metals, anticancer activity, cell line

## Abstract

Sulfonamides represent a versatile class of biologically active compounds, best known for their antibacterial activity, but increasingly investigated for their potential in oncology. Free sulfonamides themselves display cytotoxic properties; however, coordination with metal ions often enhances both selectivity and potency, while also introducing new mechanisms of action. Although numerous studies have reported sulfonamide–metal complexes with anticancer activity, a systematic overview linking biological properties to the central metal atom has been lacking. This review summarizes current research on sulfonamide complexes with transition metals and selected main-group elements, focusing on their pharmacological potential as anticancer agents. The compounds discussed include complexes of titanium, chromium, manganese, rhenium, ruthenium, osmium, iridium, palladium, platinum, copper, silver, gold, iron, cobalt, nickel, uranium, calcium, magnesium and bismuth. For each group, representative structures are presented along with cytotoxicity data against cancer cell lines, comparisons with reference drugs such as for example cisplatin, and where relevant, studies on carbonic anhydrase inhibition. The survey of available data demonstrates that many sulfonamide–metal complexes show cytotoxic activity comparable to or greater than existing chemotherapeutic agents, while in some cases exhibiting reduced toxicity toward non-cancerous cells. These findings highlight the promise of sulfonamide–metal complexes as a fertile area for anticancer drug development and provide a framework for future design strategies. This review covers the research on anti-cancer activity of sulfonamide complexes during the years 2007–2025.

## 1. Introduction

Sulfonamides are part of a broader class of biologically active compounds. Their prominence emerged from their invaluable antibacterial properties, which, during the Second World War, played a crucial role in saving countless lives. The biological activity of sulfonamides is closely linked to their molecular structure, characterized by sulfonamide and amine substituents, as well as a variety of aromatic and heteroaromatic frameworks. As of 24 August 2025, the DrugBank database lists 175 compounds classified as sulfonamides. Of these, 110 structures are either approved or in clinical trials, with 80 approved for therapeutic use. Sixty-nine structures remain under investigation, and 65 are in experimental stages. Among those approved as drugs, 16 have been withdrawn from medical practice due to health concerns. Silver salts are also present in this group as therapeutic agents; however, no records in DrugBank describe sulfonamide–metal complexes [1]. This absence does not imply a lack of such compounds in the scientific literature, where publications on the subject are in fact numerous. Despite this body of work, no comprehensive review has yet examined the biological activity of sulfonamide complexes in relation to the metal present. This article aims to address that gap by synthesizing findings from various studies and organizing them according to the type of metal forming complexes with structurally diverse sulfonamides.

Among sulfonamides with anticancer activity are sulfonamides containing various aromatic or heteroaromatic rings for example: quinazoline [2], 1,2,4-triazine [3], 1,3,5-triazine [4], thiophene [5], 3,4-dihydro-2(1H)-quinolinone [6], 1,3-oxazole [7], fluorinated pyridine [8] and many others heteroaromatic rings, as well as sulfamethoxazole-based sulfonamides [9] many chiral sulfonamides [10]. In addition, there are sulfonamide anticancer drugs that are already widely used in oncological treatment. Examples of such drugs include pazopanib [11], amsacrine (used in the treatment leukemias as well as Hodgkin’s and non-Hodgkin’s lymphomas) [12], belinostat (used in the treatment of T-cell lymphoma) [13], venetoclax (used to treat chronic lymphocytic leukemia) [14] and dabrafenib (used to treat melanoma) [15]. The sulfonamide group plays a key role in anticancer activity, but it can also act as a link between two groups that are important in increasing the activity of the compound [16].

**Figure 1:**
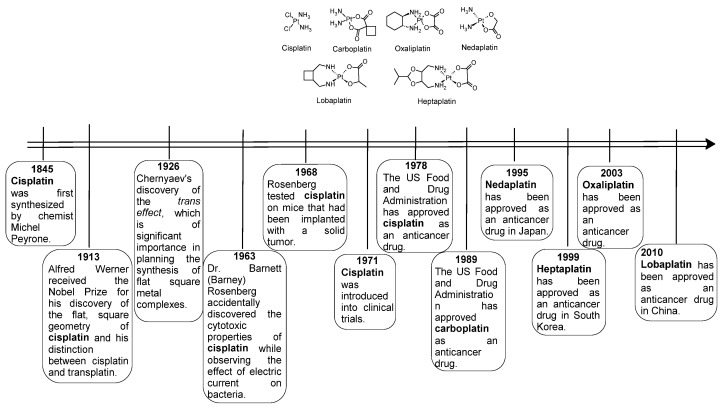
Structures of platinum complexes commonly used in medicine for cancer therapy.

In medicine, various organic compounds, acting in different ways, such as by intercalation, alkylation, etc., as well as some metal complexes, are used for widespread use in cancer treatment. The most popular anticancer drug currently used in medicine is cisplatin and its analogs: carboplatin, oxaliplatin, nedaplatin, lobaplatin and heptaplatin, shown in Figure 1. This figure also shows the history of the discovery of these drugs and their introduction into patient treatment.

The oldest platinum drug is cisplatin, obtained in 1845 by the Italian chemist Michele Peyrone. After 120 years, the cytotoxic properties of this compound were accidentally discovered by American biophysicist Barnett Rosenberg. Currently, this compound is most often used in multi-drug therapy. Cisplatin is an alkylating cytotoxic agent, which binds to DNA by covalent binding. Cisplatin has many side effects, e.g., action only on certain types of cancer, non-selectivity of the drug, adverse effects on: kidneys, nervous system, hearing, vomiting and fatigue, natural or acquired drug resistance of certain types of cancer during therapy. To reduce these side effects, new platinum drugs have been obtained and are widely used in medicine: carboplatin, oxaliplatin, nedaplatin, lobaplatin and heptaplatin. All these platinum drugs meet the rule of the so-called classical platinum drugs: platinum is present in the II oxidation state, they contain two labile anionic and two inert neutral ligands with cis geometry and the entire complexes are inert.

Over the years, many other platinum complexes deviating from these rules have been obtained and studied, e.g., complexes with trans geometry, platinum complexes on the IV oxidation state, platinum multicore complexes, encapsulation of platinum drugs in liposomal carriers and a number of complexes of other transition metals, e.g., titanium, ruthenium, osmium, gold, etc.—elements that differ in chemical properties related to their electron configuration. The most important features of the new compounds sought are cytotoxicity and selectivity. The chemical properties of the new complexes often determine different mechanisms of biological action than in the case of cisplatin and its derivatives. This could lead to a reduction in tumor resistance to the drug.

The design of new complexes with anticancer activity uses multiple organic ligands, complexing most often via a nitrogen or oxygen atom. Examples of such organic ligands are sulfonamides—compounds containing the functional group -SO_2_NR_2_. Since free sulfonamides themselves also exhibit anticancer activity [17], therefore this publication presents examples of sulfonamide complexes of d-block metals and uranium and bismuth with anti-cancer activity.

There are various mechanisms of anticancer activity of the complexes discussed. The most common mechanism is the inhibition of carbonic anhydrase IX and XII. Other mechanisms include, for example, cell cycle arrest in the S phase or G1 phase, double DNA breaks, increased levels of reactive oxygen species, interaction with proteins responsible for tumor development and high mitochondrial membrane depolarization.

The collected information on the latest sulfonamide complexes with anticancer activi-ty covers publications from the beginning of the 21st century to 2025. Databases such as Reaxys, SciFinder, and DrugBank were used for this purpose.

## 2. Sulfonamide Complexes with Metals

### 2.1. Titanium Complexes

**Figure 2:**
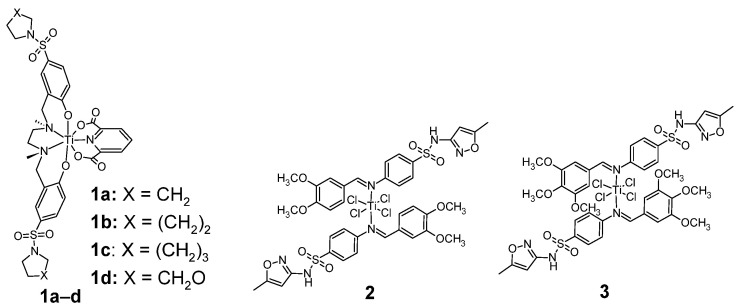
Sulfonamide complexes of titanium with antitumor activities **1**–**3.**

Sulfonamides form complexes with titanium as shown in Figure 2. Antitumor sulfonamide titanium complexes include the seven-coordinated Ti(IV) complexes **1a**–**d** [18].

The compounds **1a**–**d** were tested for anticancer activity against cell lines: Hela S3 (a derivative of the parent HeLa cell line—cervical cancer) and Hep G2 (Hepatocellular carcinoma). The anticancer activity of Ti(IV) complexes, depending on the X substituent, is shown in Table 1.

The highest antitumor activity against the HeLa S3 cell line is shown by compound **1c**, higher than cisplatin, used as a reference compound. Compound **1c** also shows the highest anti-tumor activity against the HepG2 cell line.

Another example of anticancer sulfonamide titanium complexes are the six-coordinated complexes **2** and **3** (Figure 1) [19]. The compounds were tested for anticancer activity against PC3 and HeLa cell lines (Table 2). Complex **2** was significantly more active against both cell lines than complex **3**, however, it was less active than doxorubicin.

### 2.2. Chromium(II) Complex

**Figure 3:**
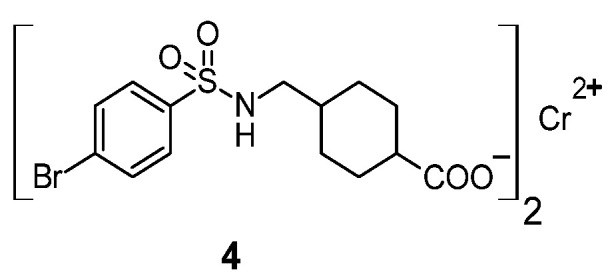
Sulfonamide complex of chromium(II) **4**.

The sulfonamide-chromium complex with anti-cancer activity is complex **4**, shown in Figure 3 [20].

The anti-cancer activity of this complex was measured by cell viability after treatment, given as a percentage (%). For the MCF-7 cell line (breast cancer), it was 45.73%, and for the HCEC cell line (human corneal epithelial cells), it was 25.40%. For the free ligand, forming this complex—the viability of MCF-7 cells was 100%, and in the case of HCEC cells was 22.97%.

### 2.3. Manganese Complexes

**Figure 4:**
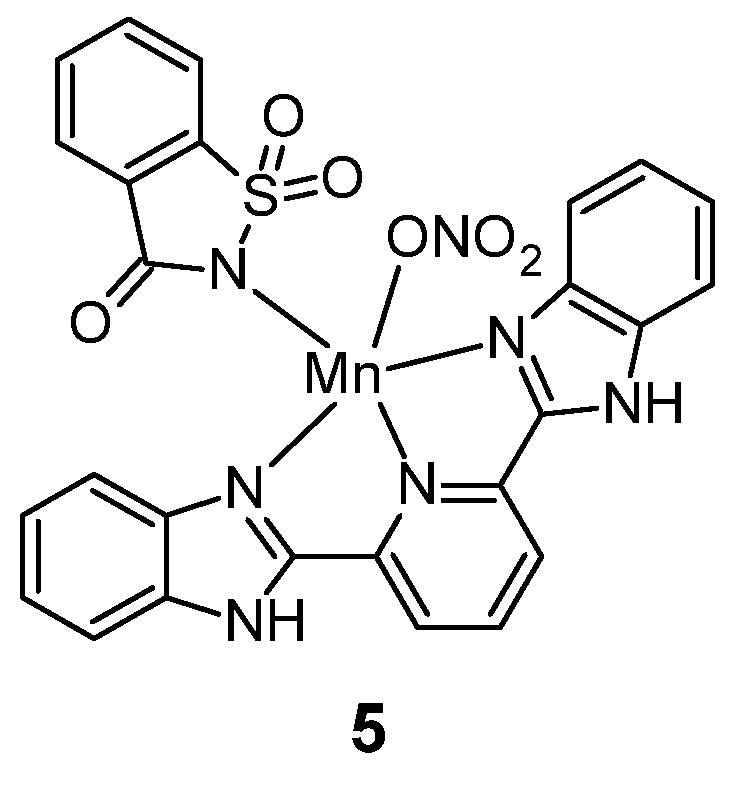
Manganese(II) complex with saccharin **5**.

Manganese(II) sulfonamide complexes include complex 5, containing saccharin and 2,6-bis(2-benzimidazolyl)pyridine (bzimpy) as a ligands (Figure 4) [21].

This complex was tested for cytotoxic activity against four cell lines: A459 (lung cancer), HT29 (colorectal adenocarcinoma), MCF-7 (breast cancer) and MCF-10A (non-tumorigenic epithelial cell line). Antitumor activity was compared with that of the bzimpy ligand and cisplatin. (Table 3).

It can be seen from Table 3 that complex **5** exhibits greater anti-cancer activity against cells of the A549 line and cells of the MCF-7 line, both in comparison to the Bzimpy ligand and in comparison to cisplatin. In contrast, 5 was less cytotoxic to the HT29 cell line, compared to the ligand and cisplatin. Both complex **5** and the free Bzimpy ligand were less cytotoxic against MCF-10A cell line than cisplatin.

A sulfonamide manganese(II) complex with an identical structure to complex **4** is also known (Figure 3). This compound showed a cell viability after treatment of 42.58% against the MCF-7 cell line and 79.86% against the normal HCEC cell line [20].

### 2.4. Rhenium Complexes

**Figure 5:**
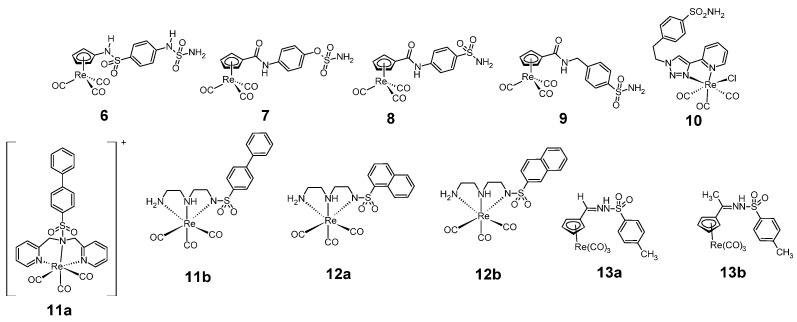
Structure of anticancer sulfonamides complexes with rhenium **6**–**13**.

Sulfonamide rhenium complexes that exhibit anticancer activity include carbonyl complexes (Figure 5). An example of such a complex is the carbonyl complexes of rhenium **6**–**9**, which have a tetrahedral structure and complexes through the aromatic system of the cyclopentadiene ring [22].

These compounds were tested for human carbonic anhydrase (hCA) inhibition. Carbonic anhydrase is a target for both diagnosis and anticancer therapy. The results of the activity of this complex on tumor-associated isoforms hCA IX and hCA XII are shown in Table 4. Inhibition was compared with acetazolamide, used as a reference compound.

This table shows that among complexes **6**–**9**, complex **7** shows the highest inhibition activity against hCA IX isosyme, while complex **9** shows the highest activity against hCA XII isosyme.

Another example of a sulfonamide carbonyl rhenium complex with anticancer activity is a six-coordinated complex **10**, containing a primary sulfonamide group, in which the metal atom is complexed via a 4-(2-pyridyl)-1,2,3-triazole group [23]. The compound was tested for carbonic anhydrase inhibition, including hCA IX and hCA XII isoforms, in the CO2 hydrase stopped-flow assay. The activity of this compound was compared with acetazolamide (Table 4).

The activity of the complex **10** against isoform hCA IX was slightly lower than activity of acetazolamide, however, against isoform hCA XII the complex **10** showed much lower activity than acetazolamide.

**Figure 6:**
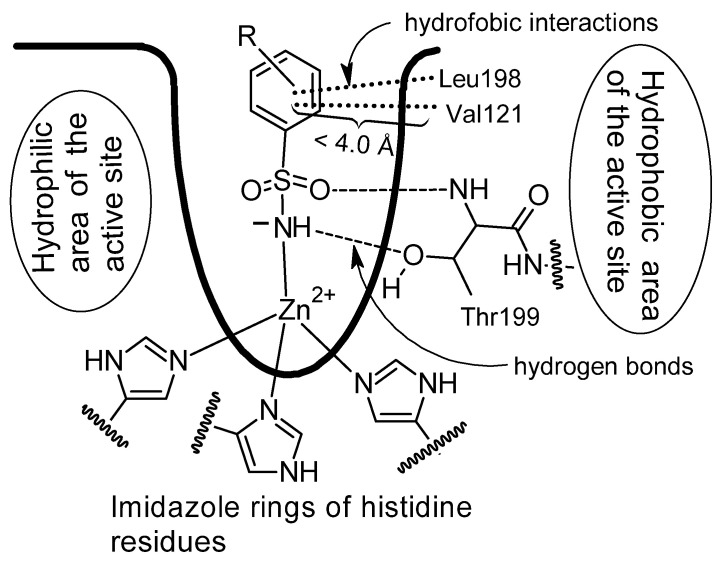
Binding of benzenesulfonamide derivatives to the carbonic anhydrase active site (Drawing made independently based on publication [24] with permission from the authors and the American Chemical Society, license number 6111840111235).

Rhenium complexes **8**–**10** are an example of primary benzenesulfonamide complexes. The Ph-SO_2_NH_2_ group is one of the most potent inhibitors of carbonic anhydrase. Its mechanism of action involves the binding of zinc ions by the deprotonated form of the sulfonamide at the lower part of the active site. In addition, hydrogen bonds are formed between the sulfonamide group and the threonine residue Thr199, as well as hydrophobic interactions between the benzene ring and the leucine residue Leu198 and valine residue Val121 in the middle part of cavity (Figure 6) [24].

Among other known compounds, there are anticancer sulfonamide rhenium six-coordinated complexes (also carbonyl), in which the rhenium atom is complexed by three nitrogen atoms, including the nitrogen atom of the sulfonamide group **11a**–**b** and **12a**–**b**. These ligands contain a biphenyl grouping [25] (**11a**–**b**) and naphthalene rings [26] (**12a**–**b**). The complexes **11a**–**b** and **12a**–**b** were tested for activity against the NCI-H292 cell line (non-small cell lung cancer cells) and compared with cisplatin (Table 5).

Compound **11a** shows slightly less anti-cancer activity than cisplatin, while compound **11b** shows much higher anti-cancer activity than cisplatin. This table shows that both complex **12a** and complex **12b** are more cytotoxic against the NCI-H292 cell line than the reference compound.

Another group of sulfonamide rhenium complexes that exhibit anti-cancer activity are sulfonamide derivatives of cyclopentadienyl imines **13a**–**b** [27]. These compounds were tested for anticancer activity against non-small cell lung cancer cell line—H1299 and compared with cisplatin (Table 6).

Compound **13a** is less active to the H1299 cell line than cisplatin. Compound **13b** is more cytotoxic against the H1299 cell line than complex **13a**, however, it is also less active than the reference compound.

**Figure 7:**
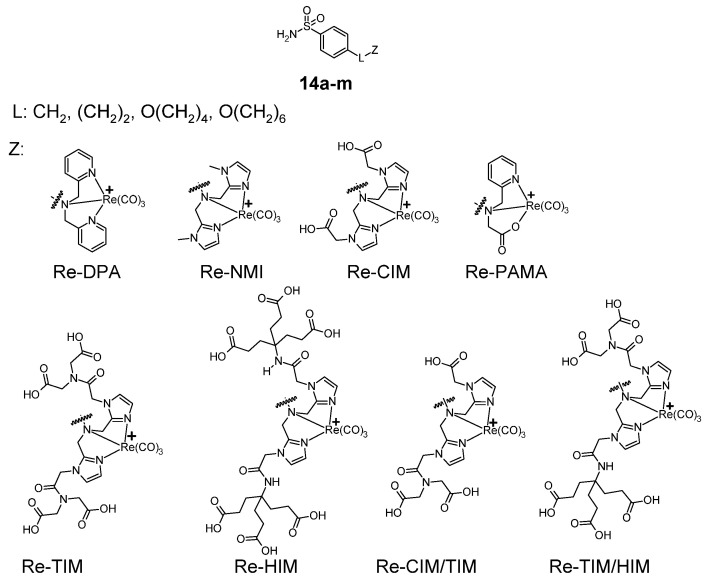
General formula of benzenesulfonamide carbonyl rhenium complexes **14a**–**m**.

A different example of sulfonamide anticancer rhenium complexes are benzenesulfonamide derivatives (**14a**–**m**), shown in Figure 7 [28]. The molecules of these compounds consist of a benzenesulfonamide group linked via a linker (symbol L in the Figure 7) to nitrogen tridentate ligands that bind the rhenium atom (Z group in the Figure 7). These complexes were tested for affinity to hCA-IX carbonic anhydrase and compared with acetazolamide (Table 7). The complex with the highest affinity for hCA-IX is 14e, showing higher affinity than acetazolamide. The lowest affinity for hCA IX was shown by compound **14a** and **14j**.

### 2.5. Iron, Cobalt, Nickel Complexes

**Figure 8:**
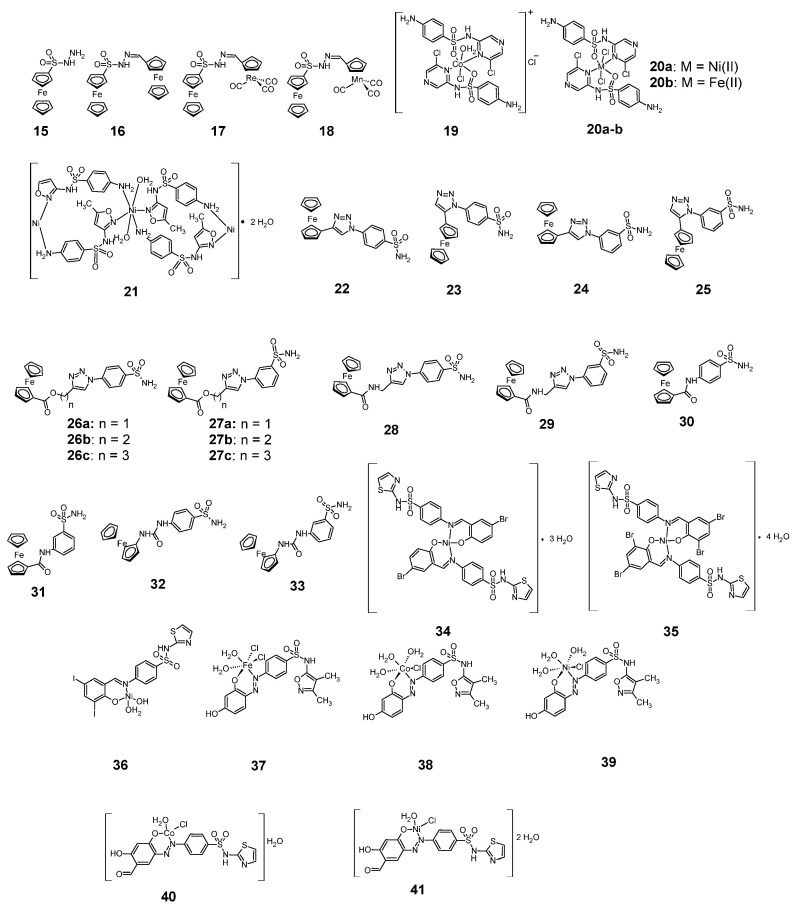
Anticancer sulfonamide complexes with iron, cobalt and nickel **15**–**41**.

Figure 8 shows sulfonamide complexes of iron, cobalt and nickel with anticancer activity. Among the anti-cancer, sulfonamide iron complexes are ferrocene complexes **15**–**18** [29].

The complexes **15**–**18** were tested for cytotoxic activity against three cancer cell lines: HCT116 (colon cancer), MCF7 (breast cancer), MDA-MB-231 (breast cancer) and against one non-cancerous BJ cell line (fibroblasts from normal foreskin). The results are shown in Table 8.

The lowest cytostatic activity, both against the cancer cell lines and against the BJ cell line, was shown by complex **15**. Against the HCT116 cell line, all complexes were less active than cisplatin. The most active complex against the HCT116 cells was complex **16**. All the complexes analyzed in this research were less cytotoxic against the non-cancerous BJ cell line than cisplatin. Complexes **15** and **16** were the least cytotoxic to the BJ cell line, while compounds **17** and **18** showed similar cytotoxicity against this cell line. In the case of the MCF-7 cell line, compound **18** was the most active, although it showed less cytotoxicity against this cell line than the reference compound. Complexes **17** and **18**, on the other hand, showed higher antitumor activity than cisplatin against the MDA-MB-231 cell line, with reduced cytotoxicity against the normal BJ cell line.

Sulfaclozine (SCZ) forms complexes **19**–**20** with iron, cobalt and nickel, shown in Figure 8 [30]. The anti-cancer activity of these complexes is shown in Table 9 and compared with sulfaclozine, as a ligand.

Among these complexes, the highest cytotoxic activity, both against MCF-7 and CaCo-2 cell lines, was shown by complex **20a**. The compound was more cytotoxic than sulfaclozine in the case of the MCF-7 cell line.

A well known compound is the sulfamethoxazole complex with nickel(II) **21**, and its cytotoxic activity against K562, HT-29 and MCF-7 cell lines is shown in Table 10 [31]. However, the compound was less cytotoxic against these three cell lines than cisplatin, used as a reference compound.

Sulfonamide complexes with iron, cobalt and nickel with an identical structure to chromium(II) complex **4** (Figure 3) exhibit cytotoxic activity, which is shown in Table 11 [20]. The most active of these compounds against the MCF-7 cell line was the nickel complex.

A. Salmon’s research group studied the anticancer activity of ferrocene complexes **22**–**33** by inhibiting the cancer-associated carbonic anhydrase enzymes IX and XII [32]. Compound 25 was the most potent inhibitor of hCA IX and hCA XII (Table 12).

Nickel complexes **34**–**36** are complexes of nickel with Schiff bases, containing a sulfonamide group [33]. Their anticancer activity is shown in Table 13. Complex **35** was the most active against the MCF-7 cell line (more active than cisplatin); however, it had similar cytotoxic properties to cisplatin against normal cells of the OEC line (normal oral epithelial).

Azo derivatives of sulfafurazole form complexes with iron **37**, cobalt **38** and nickel **39** [34]. The data in Table 14 show that all three complexes (**37**–**39**) exhibit greater cytostatic activity against A-549 and PANC-1 cell lines than the unbound sulfafurazole ligand, but this activity is weaker than that of vinblastine sulfate.

Azo derivatives of sulfathiazole form complexes **40** and **41** with Co(II) and Ni(II), respectively [35]. The cytotoxic activity of these complexes against the Hep-G2 and MCF-7 cell lines is shown in Table 15. As can be seen from this table, both complexes show greater cytotoxic activity against both cell lines than the unbound sulfathiazole ligand, while both compounds show less cytotoxic activity against the Hep-G2 cell line than cisplatin. In contrast, cobalt complex (**40**) is more cytotoxic active against the MCF-7 cell line than 5-fluorouracil.

### 2.6. Ruthenium Complexes

Among the many metals under investigation, ruthenium complexes are among a group of compounds offering hope for potential use in chemotherapy. Ru(II) and Ru(III) ions have a strong affinity for nitrogen and sulfur [36]. Three important features predispose ruthenium ions to biological studies:

(a) chemical similarity to iron due to ruthenium’s location in the same group of the periodic table, e.g., oxidation degrees and a preferential coordination number = 6,
(b) low redox potentials allowing for easy change of basic oxidation degrees under physiological conditions,
(c) relatively slow ligand exchange (a rate comparable to the rate of cell division—mitosis), which means that if ruthenium binds to a selected cell component, it will remain so bound throughout the cell life cycle [37].

**Figure 9:**
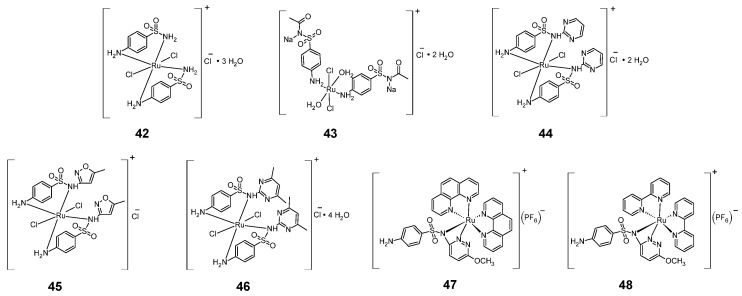
Structures of ruthenium complexes **42**–**48**.

Examples of sulfonamide complexes with ruthenium are shown in Figure 9. Among the sulfonamide ruthenium complexes with anticancer activity are chloride hexacoordinated complexes **42**–**46**. These complexes contain sulfonamides, commonly used in medicine, as ligands: sulfanilamide, sulfacetamide sodium, sulfadiazine, sulfamethoxazole and sulfadimidine [38]. These complexes were tested for cytotoxic activity against cells of the HCT-116 colon cancer line and compared with doxorubicin (Table 16). All five tested complexes showed less cytotoxicity against the HCT-116 cell line than doxorubicin. Complexes **42**–**44** were the least cytotoxic, but complex 46 was the most cytotoxic.

Another group of sulfonamide anti-cancer ruthenium complexes are sulfamethoxypyridazine complexes (**47**–**48**) [39]. The antitumor activity of these complexes against chronic myelogenous leukemia cells (K562 cell line) is shown in Table 17. Complex **48** was more active than complex **47**, however both of these complexes were much more cytotoxic than the non-sulfonamide Ru complexes: cis-[RuCl_2_(bpy)_2_] and cis-[RuCl_2_(phen)_2_].

**Figure 10:**
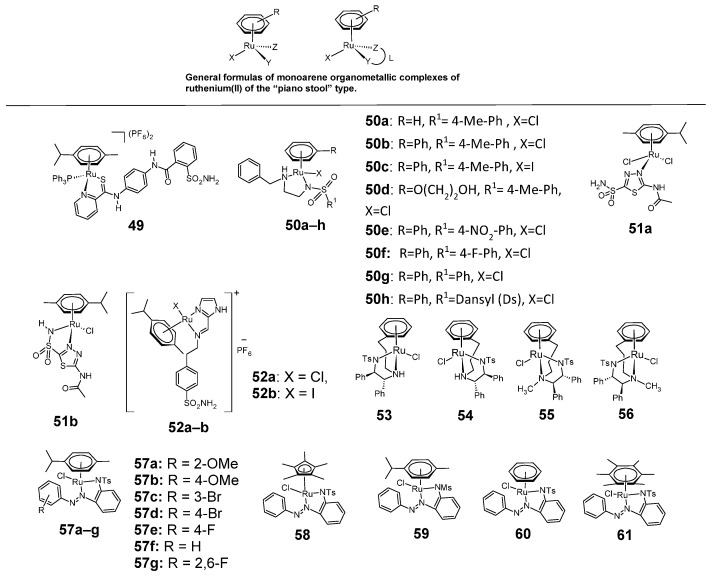
Monoarene organometallic sulfonamide complexes of ruthenium(II) of the “piano stool” type **49**–**61**.

Also of interest are organometallic compounds of ruthenium (II), the general formulas of which are shown in Figure 10. These are monoarene complexes of the “piano stool” type with pseudo-tetrahedral geometry, where the individual ligands X, Y, Z both mono- and bidentate can be varied, thus modeling their chemical properties. They are either ionic or inert compounds.

An example of such a sulfonamide ruthenium complex of the “piano stool” type is complex **49**, in which the arene, complexing the metal atom via six π electrons, is *p*-cymene [40]. The other ligands in this complex are triphenylphosphine and a sulfonamide ligand complexing through the nitrogen atom of pyridine and the sulfur atom of the thioamide group. Complex **49** was tested for cytostatic activity against SiHa cell line of cervical cancer and compared with cisplatin. This compound was more cytotoxic against SiHa cell line (IC_50_ = 1.9 ± 0.5 μM) than cisplatin (IC_50_ = 3.0 ± 1.1 μM).

Another example of sulfonamide ruthenium complexes of the “piano stool” type are compounds **50a**–**h**, in which the ruthenium atom is complexed through the nitrogen atom of the sulfonamide group, the nitrogen atom of the secondary amine group, the halogen atom (chlorine or iodine) and the aromatic ring [41]. Compounds **50a**–**h** were tested for cytotoxic activity against A2780 (ovarian cancer) and A549 (lung cancer) cell lines. The reference compound was cisplatin (Table 18).

In the case of the A2780 cell line, the greatest cytotoxic activity was exhibited by complex **50e**, which was slightly less active than cisplatin. The least active complex against the A2780 cell line was compound **50g**. However, compound **50g** was the most cytotoxic active against the A549 cell line, slightly less active than cisplatin. The least cytotoxic compound against the A549 cell line was **50h**.

Piano stool-type ruthenium sulfonamide complexes also include organometallic, chloride complexes of acetazolamide with ruthenium **51a**–**b** (Figure 10) [42]. These complexes were tested for inhibition of the cancer-associated carbonic anhydrase isoforms hCA IX and hCA XII. The reference compound was acetazolamide (Table 19).

Both complexes **51a** and **51b** were better inhibitors of hCA IX and hCA XII isoforms than acetazolamide. Complex **51b** was a better inhibitor of both isoforms than complex **51a**. It follows that the binding of acetazolamide to the ruthenium atom enhances its ability to inhibit cancer-associated isoforms hCA IX and hCA XII. The 1,3,4-thiadiazole ring linked to a sulfonamide group also causes carbonate anhydrase inhibition by binding the zinc ion. In addition, the nitrogen atoms of the 1,3,4-thiadiazole ring form hydrogen bonds with the OH group of the T200 residue of the active site [24].

Another example of sulfonamide ruthenium complexes of the piano stool type are complexes **52a**–**b**, containing an imidazole ring [43]. The anticancer activity of these compounds under normoxia and hypoxia against MIA PaCa-2 (human pancreas ductal adenocarcinoma) and MDA-MB-231 (breast adenocarcinoma) cancer cell lines is shown in Table 20. The activity of these compounds was compared against non-cancerous cells: CHO (Chinese hamster ovary) and MDCK (Madin-Darby canine kidney). Both complexes were much less cytotoxic against normal cell lines than against tumor cells.

Also known are ruthenium complexes with chiral sulfonamide (N-Tosyl) ligands **53**–**56**. Their activity against the A2780 cell line (ovarian carcinoma) is shown in Table 21, which shows that compounds **55** and **56** were more cytotoxic than cisplatin [44].

Azo sulfonamides (*N*-tosyl and *N*-mesyl) form monoarene complexes with ruthenium **57**–**61** [45]. The cytotoxicity of these compounds against four cancer cell lines: HeLa, A549, HCT-116 and MCF-7 are shown in Table 22. As can be seen from this table, the **57g** complex was the most cytostatically active against all four cell lines. It showed greater activity than cisplatin in each case.

### 2.7. Osmium Complexes

**Figure 11:**
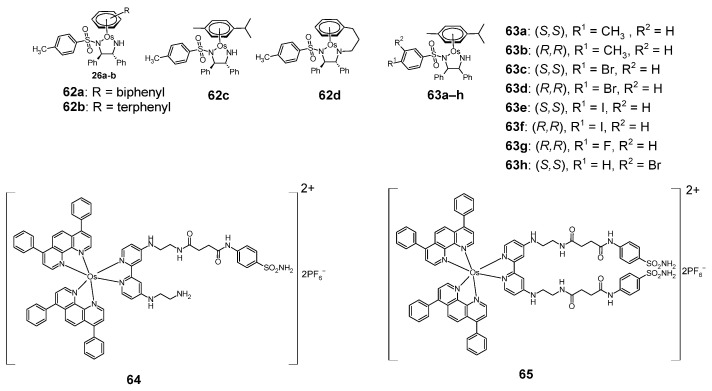
Anticancer sulfonamide complexes with osmium **62**–**65**.

Sulfonamide osmium complexes with anti-cancer activity are shown in Figure 11. Examples of such compounds are **62a**–**b** [46], **62c**–**d** [47] and **63a**–**h** [48] which also belong to the “piano stool” type complexes.

The compounds **62a**–**b** showed anticancer activity against the MRC5 (lung cancer) and A2780Cis (ovarian cancer) cell lines (Table 23).

In the case of the MRC5 cell line, the **62b** complex was more cytotoxic than cisplatin, used as a reference compound. Compound **62a** was only slightly less cytotoxic to the MRC5 cell line than cisplatin. In the case of the A2780Cis cell line, complex **62b** was also more cytotoxic than cisplatin, while complex **62a** was less cytotoxic than cisplatin.

Complexes **62c** and **62d** were tested for cytostatic activity against 7 cell lines: A2780 (ovarian cancer), A549 (lung cancer), HCT-116 (colon cancer), MCF7 (breast cancer), MCF7−TAMR-1 (tamoxifen resistant breast cancer), MCF10-A (non-tumorigenic breast cells) and MDA−MB-231 (breast cancer). The results of cytotoxic activity of **62c**–**d** complexes are summarized in Table 24. The reference compounds were cisplatin and tamoxifen. Cell lines were treated for 24 h and allowed 72 h recovery time in drug-free medium.

Table 24 shows that in the case of the A2780 cell line, compound **62d** showed greater cytotoxic activity than tamoxifen. In the case of A549, HCT-116 and MCF7 cell lines, both compounds showed less cytotoxic activity than the reference compounds, while in the case of tamoxifen-resistant MCF7-TAMR-1 cell line, compound **62d** showed only slightly less cytotoxic activity than cisplatin. Compound **62d** was also more cytotoxic against the MDA-MB-231 cell line than both reference compounds.

Table 25 shows the cytotoxic activity of **63a**–**h** complexes against cell lines: A2780 and A549. Compound **63a** was most active against both of these cell lines.

The anticancer sulfonamide osmium complexes also include compounds **64**–**65**, which are not “piano stool” type complexes [49]. These complexes were tested for phototoxic activity against two cell lines in normoxia and hypoxia: A549 and MDA-MB-231 and compared with protoporphyrin IX (PPIX)—Table 26.

These complexes (both **64** and **65**) showed greatest anti-tumor activity with 540 nm radiation. In the case of the A549 cell line in the normoxic state (at 540 nm)—complex **65** was the most active, slightly less active than protoporphyrin IX. The same cell line, being in hypoxia, was the most sensitive to Complex **64** (at 540 nm). However, this complex was less active than PPIX. Promising activity results were obtained for complex **64** at 540 nm, acting on the normoxic MDA-MB-231 cell line. This complex was only slightly less active than PPIX. For the same cell line under hypoxia, complex **64** was most active with 620 nm radiation. Complex **65**, active at 670 nm, was even more active against the MDA-MB-231 cell line under hypoxia.

### 2.8. Iridium Complexes

**Figure 12:**
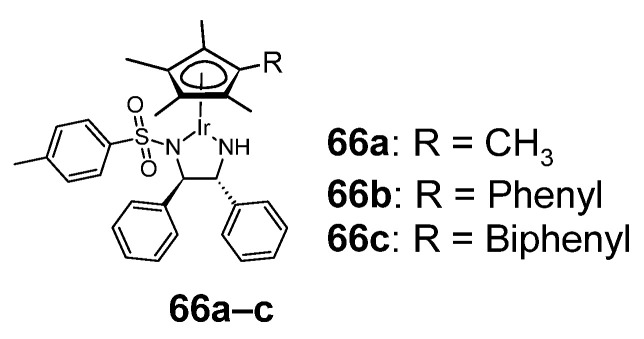
Anticancer sulfonamide complexes with iridium(III) **66a**–**c**.

An example of sulfonamide iridium(III) complexes with anti-cancer activity is cyclopentadienyl complexes **66a**–**c**, shown in Figure 12 [50]. These compounds have been studied towards 13 human cell lines (Table 27). For most cancer cell lines, compound **66c** showed the highest activity (against A2780cis and MCF-7 cell lines, it showed higher cytotoxic activity than cisplatin).

### 2.9. Palladium Complexes

**Figure 13:**
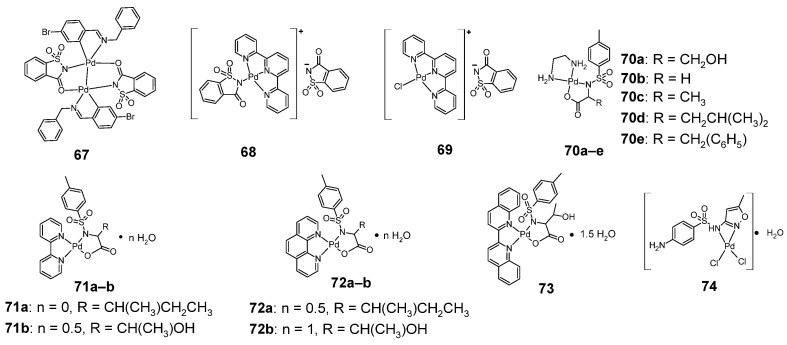
Structures of palladium-sulfonamide complexes (**67**–**74**).

Sulfonamide palladium complexes (Figure 13) with anticancer activity include palladium-saccharin complexes, for example complex **67**, containing Pd-Pd bond [51] and the cationic complexes **68** and **69**, in which saccharin is the anion [52].

K. Kazemi’s research team examined the cytotoxic activity of complex **67** against four cell lines: HeLa (cervical cancer), A549 (lung cancer), MCF-7 (breast cancer) and NIH (normal fibroblast cells). The reference compound in this study was cisplatin, sodium saccharin and free imine ligand (Table 28).

Compound **67** is more cytotoxic against A549 and MCF-7 cell lines than cisplatin, imine ligand and sodium saccharin. The cytotoxicity of compound **67** against the HeLa cell line was less than that of cisplatin, however, the palladium complex tested was much less cytotoxic against the non-cancerous NIH cell line.

E. Ulukaya’s research team tested cationic palladium-saccharin complexes (**68**–**69**) against four cell lines: A549 (lung cancer), H1299 (non-small cell lung carcinoma), PC-3 (prostatic adenocarcinoma) and CHO—chinese hamster ovary lung fibroblast (Table 29).

Compound **69** was more cytotoxic against all three cancer cell lines: A549, H1299 and PC-3 than cisplatin, but was also more cytotoxic against the non-cancer CHO cell line. Compound **68**, on the other hand, was more cytotoxic than the reference compound only to the A549 cell line, while it also showed less toxicity to non-cancerous CHO cells.

The second group of sulfonamide palladium complexes with anticancer activity are complexes of *p*-toluenesulfonyl-L-amino acid derivatives. These include complexes with ethylenediamine (**70a**–**e**) [53] and azaheteroaromatic ligands [54]: 2,2′-bipyridyl (**71a**–**b**), 1,10-phenanthroline (**72a**–**b**) and 2,2′-biquinoline (**73**). All of these complexes were tested for anticancer activity against four cell lines: HL-60, BGC-823, Bel-7402 and KB (Table 30).

In the case of the HL-60 cell line, the most active palladium complex was complex **72a**, less active than cisplatin. This compound is also the most active against the BGC-823 and Bel-7402 cell lines (however also less active than cisplatin). In contrast, in the KB cell line, the most active palladium complex is complex **72b**, which is slightly less cytotoxic than the reference compound.

Sulfamethoxazole forms a complex **74** with palladium, whose anticancer activity is shown in Table 31. The complex showed slightly lower cytotoxic activity than cisplatin against the MCF-7 cell line [31].

### 2.10. Platinum Complexes

**Figure 14:**
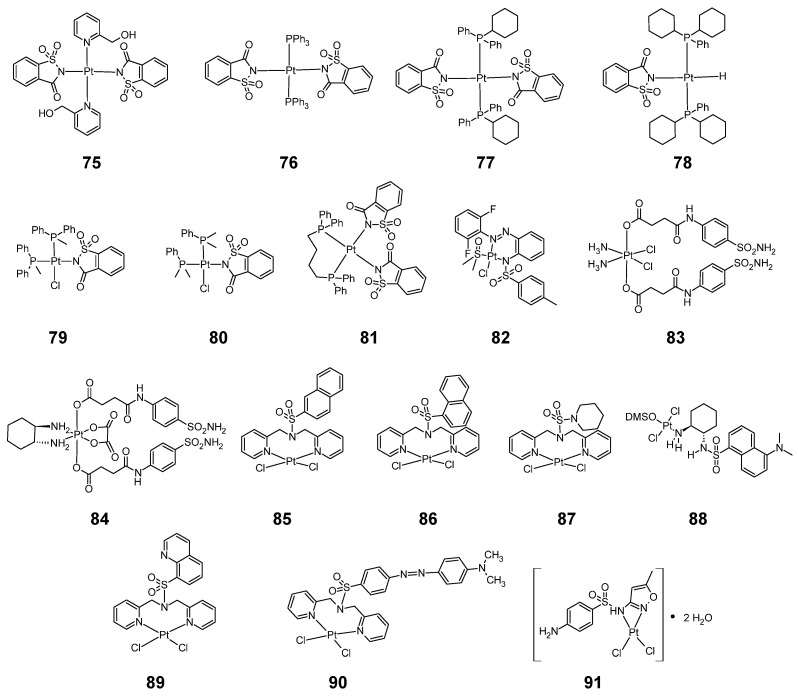
Structure of platinum complexes with sulfonamides **75**–**91**.

Platinum forms many complexes with sulfonamides with anti-cancer activity (Figure 14). An example of sulfonamides forming such complexes is saccharin, which forms complexes **75**–**81**.

The earliest studied saccharin-platinum complex was complex **75** [55]. This complex has a flat, square geometry with trans isomerism. **75** was tested for cytotoxic activity against four cell lines: A549, PC3, Hep3B, C6 and compared with carboplatin (Table 32).

As shown in Table 32, complex **75** was found to be more active against the PC3 cell line than carboplatin. For the other cell lines, complex **75** was less cytotoxic than the reference compound.

In 2018, V. T. Yilmaz’s research team published the results of cytotoxic activity of another complexes of platinum with saccharin **76**–**78** [56]. The complexes were tested for anticancer activity against: MCF-7 (breast cancer), A549 (lung cancer), DU145 (prostate cancer), HCT116 (colon cancer) cell lines and compared with cytotoxicity against normal bronchial epithelial (BEAS-2B) cells. The reference compound in these researches was cisplatin (Table 33).

Complex **77** was the most cytostatically active against all four cancer cell lines. However, it was also the most cytotoxic against non-cancerous BEAS-2B cells (more so than cisplatin). In the case of the MCF-7 cell line, all three complexes were more cytotoxic than cisplatin. In the case of the DU145 and HCT116 cell lines, compound **77** was more cytotoxic compared to cisplatin, while compound **77**’s activity against the A549 cell line was slightly less than that of cisplatin.

In 2019, Ceyd Icsel’s research team published the results of a study of the anticancer activity of two platinum-saccharin complexes: **79** and **80** [57]. The cytotoxic activity of these compounds against three cancer cell lines: A549, MCF-7, HCT-116 and one non-cancerous BEAS-2B cell line is shown in the form of three parameters in Table 34: GI_50_ (dose of 50% growth inhibition), TGI (dose of total growth inhibition) and LC_50_ (dose of 50% cell death).

Complex **79** showed slightly less cytotoxic activity against the A549 cell line than cisplatin. Good results were obtained in the MCF-7 cell line, where both complexes showed greater cytotoxic activity than cisplatin. Both complex **79** and complex **80** were less cytotoxic against the normal BEAS-2B cell line than cisplatin.

In 2023, the research team of D. I. Ugwu published the results of a cytotoxicity study of complex **81** [58]. The compound showed very good cytotoxic activity against A549 and HCT-116 cell lines, better than cisplatin. The anticancer activity against the MCF-7 cell line was slightly weaker than cisplatin, while the cytotoxicity against the non-cancerous BEAS-2B cell line was comparable to that of cisplatin (Table 35).

Sulfonamide ligands containing an azo group can forming a stable six-membered chelate ring as in the case of compound **82** (Figure 14) [59]. This complex showed cytotoxic activity against the A2780 (ovarian cancer) and A2780CP70 (cisplatin-resistant ovarian cancer) cell lines (Table 36). **82** was more cytotoxic against the A2780 cell line when DMF was used as a solubilizing solvent than when DMSO was used. The activity was slightly weaker than cisplatin, while the activity of this compound against the A2870CP70 cell line was significantly greater than cisplatin.

Other known compounds are the six-coordinate analogs (**83** and **84**) of the commonly used in oncology platinum drugs: cisplatin and oxaliplatin, containing additional axial sulfonamide ligands in trans position [60]. As shown in the Table 37, the compounds showed strong anticancer properties, under both normoxia and hypoxia conditions, against cancer cell lines: MDA-MB-231, HeLa and HepG2, more potent than cisplatin and oxaliplatin, while having reduced cytotoxic properties against normal cells: MCF-10A, LO2 and HLF.

Replacement of the ammonia molecules in the basic structure of cisplatin with molecules of the sulfonamide bipyridine ligand yields complexes **85**–**87** [61]. Compound **85** and **86** showed less cytotoxic activity against the MCF-7 breast cancer cell line than cisplatin, while complex **87** was more active against these cells (Table 38).

In addition to sulfonamide-based, planar square platinum complexes of cis geometry, compound **88** of trans geometry is also known [62]. Complex **88** had similar anticancer activity against melanoma cell line SK-MEL-5 to cisplatin. This compound, however, had higher activity than cisplatin against SK-MEL-28 cell line (Table 39).

Another sulfonamide-platinum complexes described in the 2022 publication are complexes **89**–**90**, whose cytotoxic activity against the NCl-H292 cell line is summarized in Table 40 [63]. These complexes showed higher anti-cancer activity than their free ligands. The most active after 24 h was compound **89**.

Sulfamethoxazole forms a complex **91** with platinum, whose anti-cancer activity is shown in Table 41. This compound showed less anti-cancer activity than cisplatin against three cell lines: K562, HT-29 and MCF-7 [31].

### 2.11. Copper Complexes

**Figure 15:**
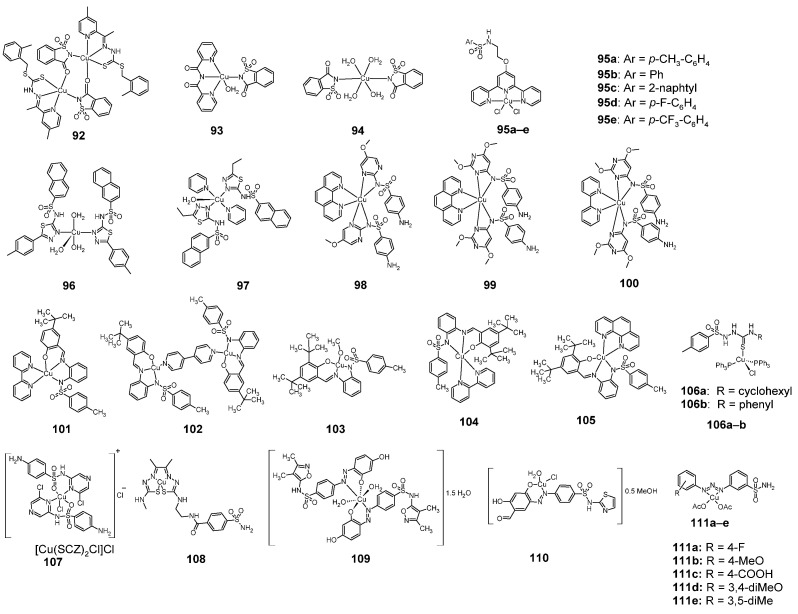
Structures of copper complexes with sulfonamides **92**–**111**.

There are many sulfonamide copper complexes with anticancer activity (Figure 15). Among the sulfonamide copper complexes with anticancer activity are complexes with saccharin, **92**–**94**.

The 2017 publication described the anticancer activity of complex **92**, containing two copper atoms, against human breast cancer cell lines: MCF-7 and MDA-MB-231 [64]. This compound was more active against both of these cell lines than tamoxifen, used as the reference compound (Table 42).

In 2021, in turn, there was a publication describing the anticancer activity of complexes **93** and **94**, each containing one copper atom [65]. Compound **93** had higher activity against MCF-7 cells than unbound saccharin and cisplatin, used as a reference compound (Table 43). However, compound **93** was also slightly more cytotoxic against the non-cancerous HEK-293 cell line than saccharin and cisplatin.

Some anticancer compounds produce reactive oxygen species (ROS) and thus induce cell death. An example of such compounds are five-coordinated complexes of copper(II) with terpyridine (**95a**–**e**) [66]. These complexes had tested their anti-breast cancer stem cell (CSC) properties. For this purpose, **95a**–**e** were tested for cytotoxic activity against the bulk breast cancer cells (HMLER) and breast CSC-enriched cells (HMLER-shEcad) cultured in monolayers (Table 44). In addition, researches were conducted for cytotoxic activity against serum-free cultures of breast CSCs, which can generate three-dimensional structures called mammospheres. All complexes were more active against HMLER cells, HMLER-shEcad cells, and HMLER-shEcad mammospheres than cisplatin and salinomycin (Table 44).

Another group of sulfonamide complexes showing anti-cancer activity are five-coordinated copper complexes with 1,3,4-thiadiazole sulfonamides (**96** and **97**) [67]. As shown in the Table 45, considering activity after 24 h—compound **97** had higher cytotoxic activity against HeLa and MW35 cell lines than cisplatin, while being less cytotoxic against the HFL1, than the reference compound.

The copper atom can also take the coordination number 6 in many complexes with azaheteroaromatic ligands, for example with pyrimidine sulfonamides (**98**–**100**) [68]. Compounds **98**–**100** were tested for cytotoxic activity against nine cancer cell lines and one non-cancer HaCat cell line and were compared with doxorubicin (Table 46).

Complex **98** was more cytotoxic against the tumor cell lines MCF-7, NCI-H460, OVCAR-3 and HT29 than doxorubicin, but was also more cytotoxic against the normal cell line HaCat. Compound **99**, in turn, showed greater activity than doxorubicin against cancerous cell lines: NCI-H460, OVCAR-3 and HT29. Its activity against HaCat cells was comparable to the reference compound.

Schiff bases derived from N-tosylbenzene-1,2-diamine form four- and five-coordinate complexes with copper(II) (**101**–**105**), showing anticancer activity (Figure 15) [69]. Table 47 shows the cytotoxic activity of compounds **101**–**105** against the SH-SY5Y, U87-MG, U373-MG cell lines and the non-cancerous MRC-5 cell line. All these compounds showed significantly greater cytotoxic activity against the SH-SY5Y neuroblastoma cell line (compound **101** was the most active) than cisplatin, used as a reference compound. Also against the U87-MG and U373-MG glioblastoma cell lines, all tested copper(II) complexes were more cytotoxic than cisplatin (compound **102** was the most active against these two cell lines).

Another sulfonamide copper complexes with potent anti-cancer properties are four-coordinated, tetrahedral biphosphine complexes of Cu(I) **106a**–**b** [70]. Both copper complexes showed greater cytotoxic activity after 12 h against the B16-F10 melanoma cell line than cisplatin, doxorubicin and their unbound ligands. The most active after 12 h was compound **106a** (Table 48).

Sulfaclozine (SCZ) forms a complex **107** with copper, which shows stronger cytotoxic properties against MCF-7 and CaCo-2 cell lines than sulfaclozine as a free ligand (Table 49) [30].

An example of copper complexes with primary sulfonamides is complex **108**, whose activity on carbonate anhydrase inhibition of hCA IX and hCA XII is shown in Table 50 [71]. This table shows that compound 108 is more active against hCA IX than all three reference compounds and the free ligand, while activity against hCA XII is similar to acetazolamide.

Compound **109** is an example of a complex of an azo derivative of sulfafurazole with copper [34]. **109** is more cytostatic active against the A-549 cell line than unbounded ligand and vinblastine sulfate (Table 51).

It is also known an analog of complex **4**, containing a copper atom instead of chromium (Figure 3). This complex showed cell viability after treatment of 80.67% for the MCF-7 cell line and 25.88% for the HCEC cell line [20].

Azo derivatives of sulfathiazole form complex **110** with Cu(II) [35]. As shown in Table 52, this compound is more cytotoxic against HepG-2 and MCF-7 cells than the unbound sulfathiazole ligand, and is more active against the MCF-7 cell line than 5-fluorouracil.

Sulfonamide derivatives of 1,3-diaryltriazene form **111a**–**e** complexes with copper [72]. These compounds were tested for their cytostatic activity against cancer cell lines: colorectal adenocarcinoma (DLD-1), cervix carcinoma (HeLa), breast adenocarcinoma (MDA-MB-231), colon adenocarcinoma (HT-29), endometrial adenocarcinoma (ECC-1), prostate cancer (DU-145 and PC-3), and compared with the normal cells: embryonic kidney (HEK-293), prostate epithelium (PNT-1A) and retinal pigment epithelium (ARPE-19) cells. The reference compound was 5-fluorouracil. The results of these studies are presented in the Table 53. In many cases, these compounds exhibited greater cytotoxic activity against cancer cell lines than 5-fluorouracil, while at the same time exhibiting less cytostatic activity against normal cell lines. For example, compound **111b** exhibited very strong cytostatic activity against the HeLa cell line (greater than 5-fluorouracil) and was much less active against normal cell lines.

### 2.12. Silver Complexes

**Figure 16:**
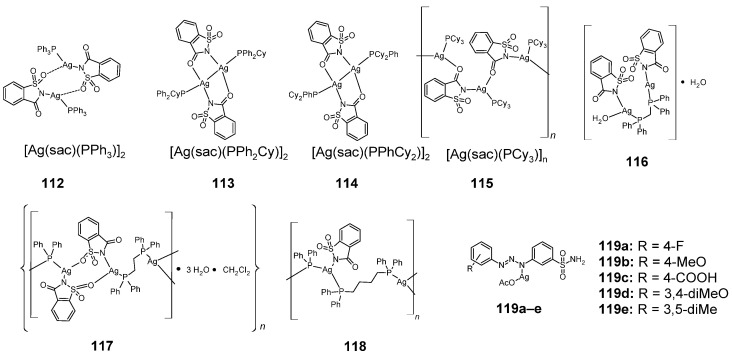
Structures of silver complexes with sulfonamides **112**–**119**.

Sulfonamide silver complexes with anticancer properties include complexes with saccharin **112**–**115** [73] and **116**–**118** (Figure 16) [74]. Compounds **112**–**115** are examples of complexes with monophosphane ligands, while compounds **116**–**118** are complexes with biphosphane ligands.

All six complexes (except for **118**) showed higher cytotoxic activity against A549 and MCF-7 cells than cisplatin and AgNO_3_. However, they were also more cytotoxic against normal WI-38 cells. Compound **113** was the most cytotoxic against these two tumor cell lines (Table 54).

Sulfonamide derivatives of 1,3-diaryltriazene form **119a**–**e** complexes with silver [72]. Cytotoxic activity against seven cancerous and three normal cell lines is presented in Table 55. Noteworthy is compound **119b**, which showed the highest cytotoxic activity against the HeLa cancer cell line among all silver complexes and higher than 5-fluorouracil (5-FU). This compound was less active against normal cell lines compared to cancer cell lines.

### 2.13. Gold Complexes

**Figure 17:**
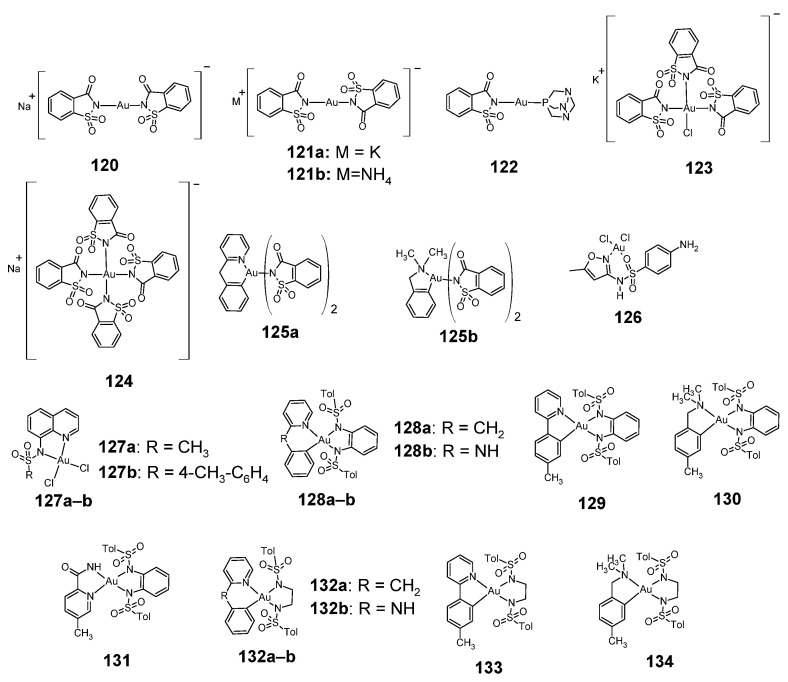
Structures of sulfonamide complexes of gold **120**–**134**.

Sulfonamide-gold complexes with anticancer activity are shown in the Figure 17. Among the sulfonamide gold complexes, gold complexes with saccharin are known, having a coordination number of 2 or 4 (**120**–**125**) [75]. Compounds **120**–**121** and **123**–**124** are in the form of salts in which the complex ion is an anion, while compound **122** is a non-ionic complex.

As shown in Table 56, complexes **120**–**124** showed less cytotoxic activity than cisplatin against cisplatin-sensitive ovarian cancer A2780/S cells; however, complexes **122** and **124** showed slightly greater activity than cisplatin against the cisplatin-resistant A2780/R cell line.

Sulfamethoxazole, an antimicrobial drug, complexes gold, forming complex **126** [76]. However, this compound showed less anti-tumor activity than doxorubicin (used as a reference compound) against the HepG-2 and HCT-116 cell lines (Table 57).

Saccharin **125a** and **125b** complexes with gold(III) were tested for cytostatic activity against the P388 cell line [77]. Compound **125a** showed an IC_50_ > 34.3 µM, while compound **125b** showed an IC_50_ > 36.0 µM.

The 8-substituted quinoline sulfonamides form 4-coordination complexes with gold(III) (**127a**–**b**) [78]. The anticancer activity of these complexes has been tested against cell lines: HBL-100, T-47D (breast cancer), HeLa (cervical cancer), SW1573 (non-small cell lung cancer), A549 (lung cancer) and WiDr (colon carcinoma). The study showed that compound **127b** exhibited greater cytotoxic activity than cisplatin against the T-47D cell line and WiDr cell lines (Table 58).

Complexes **128**–**134** are an example of complexes containing chelating bis(amidate) ligands [79]. Their anticancer activity against the P388 cell line (murine leukemia cells) is shown in Table 59, which shows that complex **131** was the most active (and also more active than cisplatin).

### 2.14. Group 10 Metal Complexes (Zinc, Cadmium and Mercury)

**Figure 18:**
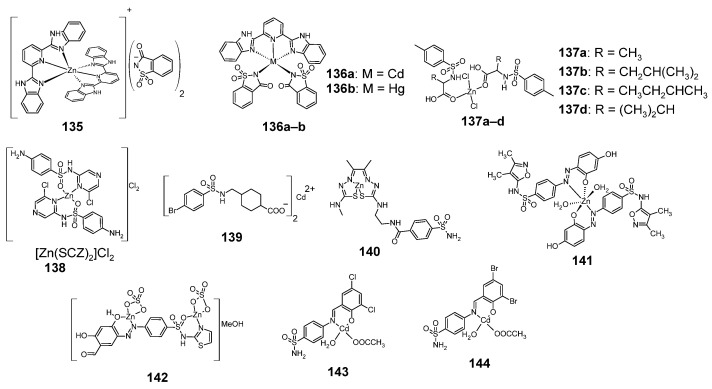
Structures of complexes of sulfonamides with zinc, cadmium and mercury **135**–**144**.

The structures of sulfonamide complexes with group 10 metals (zinc, cadmium and mercury), are shown in Figure 18. Zn, Cd and Hg form complexes with saccharin (**135** and **136a**–**b**), showing cytotoxic activity. In the case of zinc complex **135**, saccharin forms an anion, while in the case of complexes **136a**–**b** saccharin is a ligand [80].

**135** and **136a**–**b** showed cytotoxic activity against cell lines A549 (lung cancer), MCF-7 (breast cancer) and HT29 (colon cancer), as shown in Table 60. The toxicity of these complexes was compared towards normal human breast epithelial MCF10A cells.

Complex **135** (zinc complex) showed much greater cytotoxic activity than cisplatin against tumor cells: A549 and MCF-7, however it also showed higher cytotoxic activity against normal cells MCF10A than cisplatin. In the case of the HT29 cell line, compound **135** was also the most active of the complexes tested; however, it was less cytotoxic against these cells than cisplatin.

Zinc forms also **137a**–**d** complexes with sulfonamides, containing a carboxyl group [81]. The cytotoxic activity of the **137a**–**d** complexes is summarized in Table 61. The most active zinc complex against the H-157 cell line was compound **137a** (however, less cytotoxic than vincristine). This compound was also less cytotoxic to non-cancerous BHK-21 cells than vincristine.

Another example of a sulfonamide zinc complex is complex **138**, which is a complex of sulfaclozine (SCZ) with zinc [30]. This complex was more cytotoxic to the MCF-7 cell line than sulfaclozine, but was less cytotoxic to the CaCo-2 cell line (Table 62).

Compound **139**, which is a cadmium-containing analogue of complex **4** (Figure 3), showed no cytotoxic activity against the MCF-7 cell line (cell viability after treatment was 100%) [20].

Compound **140** is an analog of compound **108**, containing a zinc atom in place of a copper atom [71]. The compound shows inhibition of carbonic anhydrase hCA IX at K_i_ = 28 nM and hCA XII at K_i_ = 6.8 nM. Comparing the data in Table 50, it appears that compound **140** is less active against hCA IX and hCA XII than compound **108**.

Zinc forms a complex **141** with the azo derivative of sulfafurazole [34]. This compound shows cytostatic activity IC_50_ = 137.81 ± 10.23 µg/mL against the A-549 cell line and IC_50_ = 98.94 ± 5.78 µg/mL against the PANC-1 cell line. Thus, it is more active than the unbound sulfafurazole ligand, but less active than vinblastine sulfate and an analog containing a copper atom (compare Table 16 and Table 38).

The azo derivative of sulfathiazole forms a complex **142** with zinc [35]. The compound shows cytotoxic activity against the HepG-2 cell line at IC_50_ > 658.45 µM and against the MCF-7 cell line at IC_50_ = 30.28 µM. The complex is therefore more cytotoxic against both of these cell lines than the unbound ligand (IC_50_ > 1236.30 µM against both cell lines) and more cytotoxic against the MCF-7 cell line than 5-fluorouracil, with IC_50_ = 215.26 µM.

Sulfanilamide Schiff base derivatives form a complex with cadmium **143** and **144** [82]. The results of cytotoxicity tests on the HeLa cell line showed that complex **143** is more active than the free sulfanilamide ligand (complete inhibition of cell growth using this complex was observed at a concentration of 48.1 μg/mL, while using the ligand, this concentration was 57.1 μg/mL). Comparing the analogue of complex **143**, containing bromine atoms instead of chlorine (**144**), with its ligand, a similar correlation can be observed—complex **144** (showing complete inhibition of HeLa cell growth at a concentration of 22.5 μg/mL) is more cytostatically active than its free ligand (showing complete inhibition of HeLa cell growth at a concentration of 39.8 μg/mL).

### 2.15. Complexes of Metals Outside the d Block of the Periodic Table

**Figure 19:**
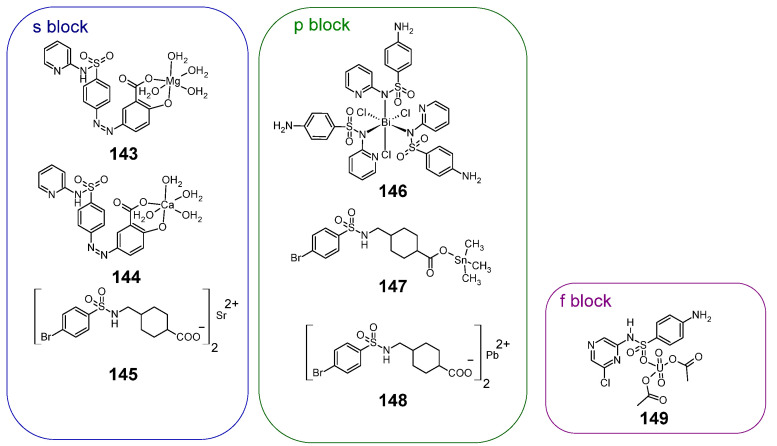
Structures of sulfonamide complexes with non-d-block metals with anti-cancer activity **145**–**151**.

Sulfonamides are also known to complex with metals outside the d block of the periodic table. Their structures are shown in Figure 19.

#### 2.15.1. Metal Complexes of the s-Block

Examples of s-block sulfonamide metal complexes are magnesium and calcium complexes, **145** and **146**, respectively [83]. The calcium complex (**146**) showed slightly greater anti-cancer activity against colon cancer cells than doxorubicin, used as a reference compound (Table 63).

It is also known strontium complex **147**, which is an analogue of chromium complex **4** [20], but it wasn’t active against MCF-7 cell line (Cell viability after treatment was 100%).

#### 2.15.2. Metal Complexes of the p-Block

The antimicrobial drug, sulfapyridine, forms a six-coordinated complex **148** with bismuth(III) [84]. Complex **148** with IC_50_ = 44 μM showed greater cytotoxic activity against the K562 leukemia cell line than the free sulfapyridine ligand used as a reference compound, with IC_50_ > 100 μM.

The cytotoxic activity (as a cell viability after treatment) of the tin (**149**) and lead (**150**) complex is shown in Table 64 [20]. Tin complex (**149**) was more cytotoxic than the lead complex, **150** (which wasn’t active) and then uncomplexed sufonamide.

#### 2.15.3. Uranium Complex

Sulfaclozine forms complex **151** with uranium, occurring as a uranyl ion [85]. The cytotoxic activity of **151** against Caco-2 cell line is shown in Table 65. The activity of this complex was compared with three reference compounds: carbimazole, 6-mercaptopurine and the free ligand sulfaclozine. Compound **151** is more cytotoxic against this cell line compared to the three given reference compounds.

## 3. Conclusions

Sulfonamides form many complexes with anti-cancer properties with metals: Ti, Mn, Re, Fe, Ru, Os, Ir, Pd, Pt, Cu, Ag, Au, Zn, Cd, Hg, U and Bi. The sulfonamide group can complex the metal atom via a nitrogen atom or an oxygen atom, or it may not participate in complexation (in this case, the metal atom is complexed by other structural elements of the molecule, containing donor atoms). The most popular type of sulfonamide complexes with metals such as rhenium, ruthenium, iridium, and osmium are “piano stool” complexes. The examples of these compounds presented here show that sulfonamide-metal complexes exhibit high anticancer activity, often greater than the reference compounds commonly used in medicine to treat cancer. Modification of the structure of sulfonamide ligands alters the cytotoxic activity of the complexes. One of the most popular sulfonamide ligands in anti-cancer complexes is saccharin. Some of the described complexes, such as for example **66c**, **82**, **122**, **124** show cytotoxic activity against cell lines resistant to some commonly used anticancer drugs (cisplatin). Many sulfonamide metal complexes, especially rhenium complexes, are involved in the inhibition of cancer-related carbonic anhydrase hCA IX and hCA XII. Most often, these are complexes of primary benzenesulfonamides. The described heterometallic complexes showed higher activity than complexes containing one metal atom—comparing complexes **15** and **16**, which contain only iron, with complexes **17** and **18** (which contain—in addition to iron—another metal), it can be concluded that the addition of another metal to the complex molecule results in increased cytotoxicity against the MDA-MB-231 cell line, but also increased cytotoxicity against the normal BJ cell line. The results of these studies may be helpful in developing new sulfonamide complexes with metals to enhance anti-cancer activity against specific cell lines. Complexes **64** and **65** as an effective drug for photodynamic therapy in oncology. Their ability to make phototoxicity in the visible and near-infrared regions of electromagnetic radiation, as well as under hypoxic conditions, carry a promise for targeted anticancer therapy. Additional axial sulfonamide ligands in cisplatin and carboplatin (compounds **83** and **84**) positively affect the activity of known platinum drugs (increasing cytotoxicity against cancerous cells, while decreasing cytotoxicity against healthy cells). Comparing the anti-cancer activity of complexes of the same ligand but with different metals, it can be deduced that swapping its metal atom for another has a major effect on activity. For example, comparing complexes of azo derivatives of sulfathiazole with various metals (copper, cobalt, nickel and zinc), **40**, **41**, **110**, **142** it can be deduced that the most cytostatic active against MCF-7 and HepG-2 cell lines is the cobalt(II) complex (**40**). However, when comparing the cytotoxic activity of carboxylate sulfonamide complexes (analogues of chromium complex **4**) with various metals based on cell viability parameters, it can be concluded that the manganese complex (an analogue of complex **4** containing manganese instead of chromium) is the most active of these compounds. Optically active sulfonamide complexes differ in their anticancer activity depending on the enantiomer, as can be seen in the example of chiral ruthenium complexes **53**–**54**.

## Figures and Tables

**Table 1:** Anticancer activity of complexes **1a**–**d**.

Compound	IC_50_ (μM)	
	Hela S3	Hep G2
**1a**	3.1 ± 0.2	9.3 ± 0.2
**1b**	0.9 ± 0.1	1.8 ± 0.2
**1c**	0.5 ± 0.1	1 ± 0.1
**1d**	19 ± 4	Non-toxic
Cisplatin	2.0 ± 0.3	4.8 ± 1.2

**Table 2:** Anticancer activity of complexes **2** and **3**.

Compound	IC_50_ (μM)	
	PC3 (Prostate Cancer)	HeLa (Cervical Cancer)
**2**	32.6 ± 0.5	36.75 ± 1.0
**3**	>100	>100
Doxorubicin	0.912 ± 0.120	3.10 ± 0.2

**Table 3:** Anticancer activity of complex **5**.

Compound	IC_50_ (μM)			
	A549	HT29	MCF-7	MCF-10A
**5**	1.60 ± 0.03	89.50 ± 2.52	3.50 ± 0.20	>100
Bzimpy ligand	8.42 ± 0.37	19.22 ± 2.84	25.33 ± 0.84	>100
Cisplatin	5.21 ± 0.18	11.25 ± 1.35	10.57 ± 0.39	13.80 ± 2.36

**Table 4:** Inhibition data of complexes **6**–**10** (AZA = acetazolamide).

CA Isozymes	K_i_ [nM]					
	6	7	8	9	10	AZA
hCA IX	43	3.7	5.2	7	29.7	25
hCA XII	6.7	4.5	6.9	4.4	45.5	5.7

**Table 5:** Anticancer activities of **11a**–**b** and **12a**–**b**.

NCI-H292 Cell Line	
Compound	IC_50_ [µM]
**11a**	97.72
**11b**	39.91
**12a**	17.33
**12b**	82.54
Cisplatin	88.18

**Table 6:** Antitumor activity of compounds **13a**–**b** against the H1299 cell line.

Compound	H1299 Cell Line IC_50_ [μM]
**13a**	37.5 ± 6.6
**13b**	24.3 ± 8.3
Cisplatin	12.8 ± 5.6

**Table 7:** Competitive Binding of complexes **14a**–**m** to Hypoxic CA-IX Expressing HeLa Cell.

Compound	L	Z	IC_50_ [nM]
Acetazolamide			7
**14a**	(CH_2_)_2_	Re-DPA	116
**14b**	(CH_2_)_2_	Re-PAMA	28
**14c**	(CH_2_)_2_	Re-NMI	53
**14d**	(CH_2_)_2_	Re-CIM	4
**14e**	(CH_2_)_2_	Re-CIM/TIM	3
**14f**	(CH_2_)_2_	Re-TIM	9
**14g**	(CH_2_)_2_	Re-TIM/HIM	44
**14h**	(CH_2_)_2_	Re-HIM	109
**14i**	CH_2_	Re-CIM	51
**14j**	CH_2_	Re-TIM	116
**14k**	O(CH_2_)_4_	Re-TIM	43
**14l**	O(CH_2_)_4_	Re-HIM	35
**14m**	O(CH_2_)_6_	Re-HIM	33

**Table 8:** Antitumor activity of compounds **15**–**18**.

	IC_50_ [μM]			
Compound	Cancer Cell Lines			Non-Cancer Cell Line
	HCT116	MCF7	MDA-MB-231	BJ
**15**	>100	>100	>100	>100
**16**	55 ± 9	42 ± 7	68 ± 10	>100
**17**	64 ± 11	63 ± 7	27 ± 2	63 ± 6
**18**	56 ± 16	33 ± 4	24 ± 2	62 ± 3
Cisplatin	21 ± 3	9.5 ± 1.5	29 ± 4	25 ± 5

**Table 9:** Antitumor activity of **19**–**20**.

Compound	IC_50_ (µg/mL)	
	MCF-7	CaCo-2
SCZ ligand	215.24 ± 0.67	97.6 ± 0.45
**19**	54.23 ± 0.52	190.1 ± 0.30
**20a**	45.62 ± 0.28	106.87 ± 0.34
**20b**	284.25 ± 0.31	362.9 ± 0.41

**Table 10:** Anticancer activity of **21**.

Compound	IC_50_(µM)		
	K562 (Chronic Myelogenous Leukemia)	HT-29 (Colon Adenocarcinoma)	MCF-7 (Breast Cancer)
**21**	51.06	44.21	42.34
Cisplatin	4.10	8.00	7.61

**Table 11:** Cytotoxic activity of analogs of complex **4** containing iron, cobalt and nickel atoms.

Compound	Cell Viability After Treatment (%)	
	MCF-7	HCEC
Iron analogue of complex **4**	100	100
Cobalt analogue of complex **4**	52.92	41.27
Nickel analogue of complex **4**	47.52	58.11

**Table 12:** CA inhibition data for compounds **22**–**33** against hCA IX and XII.

Compound	K_i_ (nM)	
	CA IX	CA XII
**22**	85	-
**23**	65	-
**24**	33.1	18.8
**25**	5.9	6.8
**26a**	80	9.2
**26b**	81	8.0
**26c**	7.9	6.8
**27a**	9.6	7.9
**27b**	9.1	7.3
**27c**	8.1	7.0
**28**	75.5	8.7
**29**	136	26.2
**30**	76.2	9.1
**31**	186	31.5
**32**	137	21.1
**33**	82.3	21.4

**Table 13:** Anticancer activity of nickel complexes **34**–**36**.

Compound	IC_50_ (μg/mL)	
	MCF-7	OEC
**34**	11.3 ± 1.1	62.23 ± 3.11
**35**	5.04 ± 0.6	33.59 ± 1.68
**36**	365 ± 24.6	>100
Cisplatin	5.71 ± 0.7	32.68 ± 2.74

**Table 14:** Anticancer activity of **37**–**39**.

Compound	IC_50_ (µg/mL)	
	A-549 (Lung Cancer)	PANC-1 (Pancreatic Cancer)
**37**	154.73 ± 11.48	102.03 ± 5.95
**38**	248.38 ± 14.65	185.81 ± 9.73
**39**	176.48 ± 10.72	120.84 ± 6.28
Ligand	466.25 ± 17.52	360.61 ± 14.63
Vinblastine sulfate	24.6 ± 0.65	4.68 ± 0.65

**Table 15:** Anticancer activity of **40**–**41**.

Compound	IC_50_ (µM)	
	Hep-G2 (Hepatocellular Carcinoma)	MCF-7 (Breast Cancer)
Ligand	>1236.30	>1236.30
**40**	197.45	26.41
**41**	562.00	224.80
Cisplatin	40.76	-
5-fluorouracil	-	215.26

**Table 16:** Cytotoxic activities against HCT-116 cell line for the Ru(III) complexes **42**–**46**.

Compound	IC_50_ [μg]
	HCT-116 Cell Line
**42**	>50
**43**	>50
**44**	>50
**45**	24.5
**46**	10.3
Doxorubicin	0.471

**Table 17:** Cytotoxic activities against K562 cell line for the complexes **47**–**48**.

Compound	IC_50_ (μM)
	K562 Cell Line
**47**	3.80 ± 0.19
**48**	2.00 ± 0.10
*cis*-[RuCl_2_(bpy)_2_]	>100
*cis*-[RuCl_2_(phen)_2_]	>100

**Table 18:** Cytotoxic activity of complexes **50a**–**h**.

Compound	IC_50_ [μM]	
	A2780 Cell Line	A549 Cell Line
**50a**	8.32 ± 0.54	28.8 ± 2.6
**50b**	11.25 ± 0.08	13.5 ± 1.4
**50c**	18.4 ± 1.2	32.2 ± 0.7
**50d**	14.25 ± 0.06	16.1 ± 2.4
**50e**	3.57 ± 0.98	29.8 ± 1.1
**50f**	5.6 ± 0.5	13.7 ± 0.1
**50g**	>50	4.1 ± 1.3
**50h**	39.4 ± 3.4	38.5 ± 1.9
Cisplatin	1.2 ± 0.02	3.1 ± 0.1

**Table 19:** Inhibition of isoforms hCA IX and hCA XII by acetazolamide and **51a**–**b**.

Compound	K_I_ [nM]	
	hCA IX	hCA XII
**51a**	3.8	0.52
**51b**	0.63	0.04
Acetazolamide	25.2	5.7

**Table 20:** Anticancer activity of **52a**–**b**.

Compound	IC_50_ (μM)				
	Normoxia (~12% O_2_)				Hypoxia (~1.5% O_2_)
	CHO	MDCK	MIA PaCa-2	MDA-MB-231	MDA-MB231
**52a**	>200	>200	22.5 ± 1.5	38.3 ± 1.5	27.9 ± 1.0
**52b**	>200	>200	17.1 ± 1.0	39.6 ± 3.2	17.7 ± 1.8

**Table 21:** Anticancer activity of **53**–**56** against A2780 cell line.

Compound	GI_50_ (μM)
	A2780 Cell Line
**53**	5.5 ± 0.5
**54**	13.7 ± 0.4
**55**	1.2 ± 0.3
**56**	1.8 ± 0.2
Cisplatin	2.0 ± 0.2

**Table 22:** Anticancer activity of **57**–**61**.

Compound	IC_50_ (µM)			
	HeLa	A549	HCT-116	MCF-7
**57a**	10.3 ± 1.3	n.d.	n.d.	n.d.
**57b**	3.3 ± 0.4	n.d.	n.d.	n.d.
**57c**	3.1 ± 0.4	n.d.	n.d.	n.d.
**57d**	7.0 ± 0.5	n.d.	n.d.	n.d.
**57e**	4.1 ± 0.7	13.8 ± 2.0	11.2 ± 2.8	21.9 ± 2.0
**57f**	3.6 ± 0.5	12.6 ± 1.4	9.8 ± 0.7	9.2 ± 0.7
**57g**	1.7 ± 0.4	2.5 ± 0.3	1.3 ± 0.1	8.5 ± 1.8
**58**	>100	n.d.	n.d.	n.d.
**59**	9.3 ± 1.0	n.d.	n.d.	n.d.
**60**	13.0 ± 1.4	n.d.	n.d.	n.d.
**61**	>100	n.d.	n.d.	n.d.
Cisplatin	7.0 ± 1.5	11.7 ± 2.0	16.0 ± 2.3	12.5 ± 0.9

n.d. = not determined.

**Table 23:** Anticancer activities of **62a**–**b**.

Compound	IC_50_ [μM]	
	MRC5 Cell Line	A2780Cis
**62a**	15.1 ± 0.5	25.9 ± 0.7
**62b**	9.7 ± 0.2	10.4 ± 0.5
Cisplatin	13.5 ± 0.9	11.5 ± 0.2

**Table 24:** Anticancer activities of **62c**–**d** against seven cell lines (N.D. = not determined).

Compound	IC_50_ [μM]						
	A2780	A549	HCT-116	MCF7	MCF7−TAMR-1	MCF10-A	MDA−MB-231
**62c**	15.5 ± 0.5	21.1 ± 0.3	37 ± 1	11.0 ± 0.3	N.D.	N.D	15 ± 1
**62d**	10.5 ± 0.1	14.1 ± 0.3	36.4 ± 0.2	8.1 ± 0.2	9 ± 1	30.9 ± 0.4	9.1 ± 0.9
Cisplatin	1.2 ± 0.3	3.2 ± 0.1	5.2 ± 0.3	6.6 ± 0.2	6.0 ± 0.7	6 ± 1	9.6 ± 0.4
Tamoxifen	12.4 ± 0.1	13.5 ± 0.1	19 ± 3	5.9 ± 0.4	14.5 ± 0.1	25.0 ± 0.2	12.9 ± 0.2

**Table 25:** Anticancer activity of **63a**–**h**.

Compound	IC_50_ (μM)	
	A2780 (Ovarian Cancer)	A549 (Lung Cancer)
**63a**	15.2 ± 0.5	21.1 ± 0.3
**63b**	15.5 ± 0.5	31 ± 1
**63c**	31 ± 2	29.5 ± 0.5
**63d**	27.4 ± 0.6	33 ± 0.4
**63e**	27.5 ± 0.8	32 ± 0.4
**63f**	29 ± 3	-
**63g**	17 ± 1	32 ± 2
**63h**	27 ± 2	21.1 ± 0.3

**Table 26:** Phototoxic activity of compounds **64** and **65** against A549 and MDA-MB-231 cell lines (in normoxia and hypoxia) at four wavelengths of radiation.

Compound	IC_50_ (540 nm) [μM]	IC_50_ (620 nm) [μM]	IC_50_ (670 nm) [μM]	IC_50_ (740 nm) [μM]
**A549 cell line (Normoxia 20%)**				
**64**	8.1±1.9	>30	26.2 ± 5.4	22.5 ± 3.8
**65**	4.4 ± 0.1	10.4 ± 0.7	9.9 ± 0.9	7.0 ± 0.1
PPIX	0.3 ± 0.1	2.7 ± 0.1	3.0 ± 0.1	>100
**A549 cell line (Hipoxia 2%)**				
**64**	14.6 ± 3.1	>30	>100	>100
**65**	>30	>30	>100	>100
PPIX	0.7 ± 0.2	2.2 ± 0.9	3.5 ± 0.3	>100
**MDA-MB-231 cell line (Normoxia 20%)**				
**64**	1.2 ± 0.1	6.2 ± 2.8.	3.9 ± 0.5	3.6 ± 0.4
**65**	2.2 ± 0.1	2.6 ± 0.5	3.0 ± 1.2	0.7 ± 0.2
PPIX	0.8 ± 0.4	1.0 ± 0.5	0.7 ± 0.3	-
**MDA-MB-231 cell line (Hipoxia, 2%)**				
**64**	>10	9.2 ± 3.0	12.0 ± 1.0	>30
**65**	>30	>30	4.6 ± 0.6	>30
PPIX	0.6 ± 0.1	1.8 ± 0.4	0.4 ± 0.1	-

**Table 27:** Anticancer activity of sulfonamide complexes with iridium **66a**–**c** (N.D. = not determined).

Cell Lines	IC_50_ (μM)			
	66a	66b	66c	Cisplatin
**A2780 (ovarian cancer)**	20.9 ± 0.7	14 ± 2	10.2 ± 0.6	1.2 ± 0.3
**A2780cis (ovarian, cisplatin-resistant)**	17 ± 2	18 ± 1	6.3 ± 0.8	13.4 ± 0.3
**A549 (lung cancer)**	38 ± 2	25.2 ± 0.3	17 ± 3	3.2 ± 0.1
**HCT116 (colorectal cancer)**	40.3 ± 0.7	58.2 ± 0.9	15.0 ± 0.1	5.2 ± 0.3
**HCT116-p21-/- (colorectal cancer, p21 knockout)**	62.4 ± 0.3	N.D.	N.D.	9.2 ± 0.5
**HCT116-p53-/- (colorectal cancer, p53 knockout)**	63 ± 2	N.D.	N.D.	36.7 ± 0.3
**MCF10-A (breast, non-tumorigenic)**	42 ± 3	N.D.	N.D.	6 ± 1
**HOF (ovarian, non-tumorigenic)**	19.0 ± 0.1	26.8 ± 0.6	15.0 ± 0.7	10.2 ± 0.7
**MCF7 (breast cancer)**	14.6 ± 0.2	8 ± 1	3.7 ± 0.7	6.6 ± 0.4
**MCF7-TAMR1 (breast, tamoxifen-resistant)**	17.8 ± 0.6	N.D.	N.D.	6.0 ± 0.7
**MRC5 (lung, non-tumorigenic)**	17.3 ± 0.4	11.9 ± 0.9	5.9 ± 0.2	12.8 ± 0.5
**OE19 (esophageal cancer)**	>50	33.2 ± 0.5	12.9 ± 0.3	9 ± 1
**PC3 (prostate cancer)**	35.1 ± 0.6	18.8 ± 0.2	12 ± 1	4.1 ± 0.5

**Table 28:** Anticancer activities of **67**.

Compound	IC_50_ [µM]			
	HeLa	A549	MCF-7	NIH
sodium saccharin	>200	>200	>200	-
Ligand	>200	93.8 ± 4	>200	-
**67**	30.4 ± 2	23.1 ± 1	22.2 ± 1	165 ± 1
Cisplatin	17.6 ± 1	24.3 ± 1	26.3 ± 1	38.67 ± 4

**Table 29:** Cytotoxic activities of **68** and **69**.

Compound	IC_50_ [µM]			
	A549	H1299	PC-3	CHO
**68**	2.3	8.6	9.6	5.8
**69**	2.1	1.8	1.8	1.9
Cisplatin	23.1	3.2	8.6	3.2

**Table 30:** Cytotoxic activities of palladium complexes **70**–**73**.

Compound	IC_50_ [µM]			
	HL-60 (Leukemia)	BGC-823 (Gastrocarcinoma)	Bel-7402 (Liver Carcinoma)	KB (Nasopharyngeal Carcinoma)
**70a**	26 ± 1	48 ± 2	45 ± 1	32 ± 2
**70b**	23 ± 1	38 ± 2	35.8 ± 0.9	37 ± 2
**70c**	15.9 ± 0.5	41 ± 1	33.9 ± 0.6	12 ± 1
**70d**	12.9 ± 0.9	35.8 ± 0.8	33 ± 2	15.6 ± 0.9
**70e**	12 ± 1	34 ± 2	28 ± 1	11.8 ± 0.5
**71a**	18.66 ± 1.07	23.74 ± 1.23	44.52 ± 1.43	9.28 ± 1.07
**71b**	16.76 ± 1.08	25.78 ± 1.11	40.76 ± 1.45	6.54 ± 0.97
**72a**	9.20 ± 0.97	21.70 ± 2.08	18.01 ± 1.32	6.08 ± 0.87
**72b**	12.46 ± 1.05	23.45 ± 1.32	38.98 ± 2.08	5.98 ± 0.65
**73**	16.54 ± 1.21	30.89 ± 1.34	30.76 ± 2.07	22.54 ± 1.23
Cisplatin	2.89 ± 0.34	6.48 ± 0.81	8.12 ± 0.97	2.65 ± 0.33

**Table 31:** Anticancer activity of palladium complex with sulfamethoxazole (**74**).

Compound	IC_50_(µM)		
	K562	HT-29	MCF-7
**74**	7.20	10.42	8.64
Cisplatin	4.10	8.00	7.61

**Table 32:** Cytotoxic activities of **75** against four cancer cell lines.

Cell Lines		IC_50_ [µM]	IC_90_ [µM]
**A549 (lung cancer)**	**75**	117.5	192.4
	Carboplatin	70.5	>85.1
**PC3 (prostate cancer)**	**75**	72.2	95.6
	Carboplatin	85.1	>85.1
**Hep3B (liver cancer)**	**75**	35.0	82.1
	Carboplatin	6.8	28.7
**C6 (glioma)**	**75**	38.9	90.9
	Carboplatin	7.6	42.7

**Table 33:** Antitumor activities of **76**–**78**.

Compound	IC_50_ [µM]				
	MCF-7	A549	DU145	HCT116	BEAS-2B
**76**	8.4 ± 0.1	10.1 ± 1.7	14.5 ± 0.5	13.0 ± 0.7	8.1 ± 0.3
**77**	3.8 ± 0.4	4.0 ± 0.1	4.6 ± 0.1	4.8 ± 0.1	2.4 ± 0.1
**78**	17.0 ± 3.7	30.1 ± 1.0	37.7 ± 0.1	36.7 ± 0.2	27.7 ± 1.2
Cisplatin	24.0 ± 4.0	2.5 ± 0.9	9.8 ± 4.5	15.5 ± 2.3	4.6 ± 0.2

**Table 34:** GI_50_, TGI and LC_50_ values (after 48 h) for complexes **79** and **80**.

Cell Lines		79	80	Cisplatin
**A549**	**GI_50_ [µM]**	16.42 ± 0.85	24.56 ± 4.34	14.38 ± 0.47
	**TGI [µM]**	29.13 ± 1.43	>40	22.63 ± 0.38
	**LC_50_ [µM]**	>40	>40	37.77 ± 0.65
**MCF-7**	**GI_50_ [µM]**	7.38 ± 0.64	5.38 ± 0.03	10.88 ± 0.18
	**TGI [µM]**	13.40 ± 0.66	22.06 ± 0.57	15.19 ± 0.10
	**LC_50_ [µM]**	18.93 ± 0.64	38.64 ± 0.68	19.50 ± 0.01
**HCT-116**	**GI_50_ [µM]**	12.19 ± 0.26	12.72 ± 1.55	9.03 ± 1.46
	**TGI [µM]**	17.12 ± 0.23	24.39 ± 2.53	21.24 ± 1.71
	**LC_50_ [µM]**	28.18 ± 0.49	>40	34.28 ± 1.10
**BEAS-2B**	**GI_50_ [µM]**	8.52 ± 0.63	21.46 ± 0.76	3.85 ± 0.03
	**TGI [µM]**	14.06 ± 0.81	31.06 ± 0.59	6.18 ± 0.17
	**LC_50_ [µM]**	26.95 ± 2.56	>40	10.83 ± 1.24

**Table 35:** Cytotoxic activity of **81**.

Compound	IC_50_ [µM]			
	A549	MCF-7	HCT-116	BEAS-2B
**81**	7.32	16.89	6.88	6.82
Cisplatin	17.23	11.68	14.69	6.29

**Table 36:** The cytotoxic activity of **82** depending on the solubilizing solvent used.

Compound	Solubilizing Solvent	IC_50_ [µM]	
		A2780	A2870CP70
**82**	DMSO	1.0 ± 0.3	2.7 ± 0.6
	DMF	0.43 ± 0.05	0.8 ± 0.1
Cisplatin	PBS (Phosphate-buffered saline)	0.18 ± 0.07	5 ± 1

**Table 37:** Anti-cancer activity of compounds **83** and **84** against cell lines under normoxia and hypoxia.

Cell Lines	IC_50_ [μM]			
	83	84	Cisplatin	Oxaliplatin
**Normoxia (20% O_2_)**				
**MDA-MB-231 (breast cancer)**	18.0 ± 1.8	27.6 ± 2.3	14.6 ± 0.8	35.4 ± 2.0
**HeLa (cervical cancer)**	12.6 ± 1.2	41.7 ± 2.3	15.9 ± 1.3	28.1 ± 2.6
**HepG2 (hepatocellular carcinoma)**	28.6 ± 0.6	30.4 ± 0.8	17.9 ± 1.9	8.1 ± 0.8
**LO2 (hepatocyte cell line)**	158.5 ± 3.4	>200	12.0 ± 1.5	31.0 ± 5.3
**HLF (lung fibroblast)**	102.6 ± 8.6	>200	10.7 ± 2.9	23.7 ± 1.9
**MCF-10A ((mammary epithelial cell line)**	153.7 ± 3.6	>200	17.9 ± 1.9	8.9 ± 0.4
**Hipoxia (1% O_2_)**				
**MDA-MB-231**	1.9 ± 0.9	5.8 ± 2.1	21.9 ± 5.9	65.7 ± 5.3
**HeLa**	3.6 ± 0.3	15.8 ± 2.6	28.2 ± 1.7	>50
**HepG2**	8.3 ± 1.8	5.6 ± 1.5	20.2 ± 3.8	18.1 ± 2.1

**Table 38:** Antitumor activity of **85**–**87** complexes against MCF-7 cells.

Compound	IC_50_ [μM] MCF-7 Cell Line
**85**	529.3 ± 0.22
**86**	135.7 ± 0.06
**87**	16.1 ± 0.08
Cisplatin	97.86

**Table 39:** Antitumor activity of **88** against melanoma cell lines: SK-MEL-5 and SK-MEL-28.

Compound	IC_50_ [μM]	
	SK-MEL-5	SK-MEL-28
**88**	3.24 ± 1.08	5.83 ± 1.06
Cisplatin	2.89 ± 1.12	10.17 ± 1.29

**Table 40:** Antitumor activity of **89**–**90** and their ligands against NCl–H292 lung cancer cells after 24, 48 and 72 h.

Compound	IC_50_ [μg/mL]		
	24 h	48 h	72 h
**89**	37.83	15.94	9.01
Ligand of **89**	225	31.82	36.49
**90**	112.8	26.21	12.13
Ligand of **90**	612.4	17.88	13.95

**Table 41:** Anticancer activity of **91**.

Compound	IC_50_(µM)		
	K562	HT-29	MCF-7
**91**	9.67	12.12	11.37
Cisplatin	4.10	8.00	7.61

**Table 42:** Antitumor activity of **92**.

Compound	IC_50_ [μM]	
	MCF-7	MDA-MB-231
**92**	3.60	3.81
Tamoxifen	4.04	13.46

**Table 43:** Anticancer activity of **93** and **94**.

Compound	IC_50_ [μM]	
	MCF-7	HEK-293
**93**	5.62 ± 2.28	93.44 ± 12.17
**94**	18.86 ± 1.01	94.43 ± 10.18
Saccharine	46.60 ± 7.92	>100
Cisplatin	10.37 ± 3.11	>100

**Table 44:** IC_50_ values of the complexes **95a**–**e** against HMLER cells, HMLER-shEcad cells, and HMLER-shEcad mammospheres.

Compound	IC_50_ [μM]		
	HMLER	HMLER-shEcad	HMLER-shEcad Mammospheres
**95a**	0.85 ± 0.04	0.69 ± 0.12	3.44 ± 0.03
**95b**	0.85 ± 0.14	0.83 ± 0.01	2.98 ± 0.01
**95c**	0.83 ± 0.06	0.73 ± 0.04	2.63 ± 0.08
**95d**	0.83 ± 0.19	0.78 ± 0.03	2.99 ± 0.53
**95e**	0.81 ± 0.02	0.79 ± 0.18	3.34 ± 0.36
Cisplatin	2.57 ± 0.02	5.65 ± 0.30	13.50 ± 2.34
Salinomycin	11.43 ± 0.42	4.23 ± 0.35	18.50 ± 1.50

**Table 45:** IC_50_ values of **96, 97** and cisplatin on the HeLa, WM35, and HFL1 cell lines after 24, 48 and 72 h.

Cell Line	Compound	IC_50_ [μM]		
		24 h	48 h	72 h
**HeLa (cervical carcinoma)**	**96**	33.18 ± 0.19	16.36 ± 0.12	6.47 ± 0.06
	**97**	8.79 ± 0.21	4.06 ± 0.05	1.45 ± 0.09
	Cisplatin	21.03 ± 0.14	21.03 ± 0.14	2.39 ± 0.04
**MW35 (radical growth phase melanoma)**	**96**	41.35 ± 0.19	23.87 ± 0.17	15.42 ± 0.08
	**97**	13.01 ± 0.15	8.11 ± 0.13	4.66 ± 0.07
	Cisplatin	26.07 ± 0.43	11.15 ± 0.09	5.98 ± 0.03
**HFL1 (normal fibroblastic epithelial cell line)**	**96**	44.67 ± 0.51	7.38 ± 0.12	3.75 ± 0.15
	**97**	17.99 ± 1.08	5.55 ± 0.22	2.03 ± 0.04
	Cisplatin	13.22 ± 0.89	3.99 ± 0.22	1.18 ± 0.13

**Table 46:** Total Growth Inhibition (TGI) [μM] values of the copper(II) complexes **98**–**100**.

Cell Line	TGI [μM]			
	98	99	100	Doxorubicin
**U251 (glioma)**	1.4	<0.3	35.3	0.1
**UACC-62 (melanoma)**	5.1	7.8	51.2	1.4
**MCF-7 (breast cancer)**	15.4	46.4	217.0	46.0
**NCI-ADR/RES (multidrug resistant ovarian cancer)**	291.9	111.0	298.2	46.0
**786**–**0 (renal cancer)**	291.9	47.9	298.2	0.4
**NCI-H460 (lung, non-small cancer cells)**	7.9	7.1	64.8	46.0
**OVCAR-3 (ovarian cancer)**	3.3	4.7	75.9	26.5
**HT29 (colon cancer)**	6.3	4.9	129.3	46.0
**K562 (leukemia)**	35.4	32.5	298.2	11.6
**HaCat (immortal keratinocyte, non-tumor human line)**	14.0	46.1	298.2	46.0

**Table 47:** Cytotoxicity of copper(II) complexes **101**–**105** towards the SH-SY5Y, U87-MG, U373-MG and MRC-5 cell lines.

Compound	IC_50_ [μM]			
	SH-SY5Y (Neuroblastoma)	U87-MG (Glioblastoma)	U373-MG (Glioblastoma)	MRC-5 (Mortal Human MRC5 Fibroblasts)
**101**	2.63 ± 0.40	20.09 ± 0.05	14.42 ± 0.96	16.85 ± 0.57
**102**	2.85 ± 0.43	11.83 ± 1.07	12.92 ± 1.05	19.94 ± 1.72
**103**	9.06 ± 1.36	26.73 ± 5.26	17.61 ± 2.27	25.39 ± 1.32
**104**	23.55 ± 3.53	22.25 ± 0.89	19.78 ± 2.03	24.91 ± 1.26
**105**	5.57 ± 0.84	25.92 ± 1.25	22.52 ± 1.35	24.92 ± 2.28
Cisplatin	27.50 ± 4.30	>100	~150	–

**Table 48:** IC_50_ values of **106a**–**b** complexes, ligands, cisplatin and doxorubicin against B16-F10 melanoma cell line.

Compound	IC_50_ [µM] B16-F10 Cell Line			
	12 h	24 h	36 h	48 h
**106a**	14.4 ± 2.4	13.0 ± 3.7	16.5 ± 2.4	11.6 ± 1.9
Ligand of **106a**	256.1 ± 21.3	152.9 ± 13.2	62.9 ± 5.2	41.7 ± 2.0
**106b**	32.6 ± 3.1	22.8 ± 2.0	19.5 ± 1.9	18.4 ± 1.7
Ligand of **106b**	265.0 ± 22.1	112.5 ± 9.0	58.2 ± 4.1	55.9 ± 3.4
Cisplatin	132.9 ± 10.5	58.2 ± 2.3	38.4 ± 3.2	36.7 ± 2.1
Doxorubicin	288.0 ± 10.0	10.1 ± 2.1	3.1 ± 1.3	2.2 ± 1.1

**Table 49:** Anticancer activity of **107**.

Compound	IC_50_ (µg/mL)	
	MCF-7	CaCo-2
**107**	86.2 ± 0.64	23.84 ± 0.33
Sulfaclozine (SCZ)	215.24 ± 0.67	97.6 ± 0.45

**Table 50:** Enzyme inhibition for **108** and reference compounds (FTES = (4-sulfamoylphenylethyl-thioureido)-fluorescein, ABS = 4-(2-aminoethyl)-benzenesulfonamide).

Compound	K_i_ (nM)	
	CA IX	CA XII
Ligand	62	73
**108**	9.0	6.1
ABS	33	3.2
Acetazolamide (AZA)	25	5.7
FTES	24	-

**Table 51:** Anticancer activity of **109**.

Compound	IC_50_ (µg/mL)	
	A-549 (Lung Cancer)	PANC-1 (Pancreatic Cancer)
**109**	12.26 ± 0.73	13.43 ± 0.81
Ligand	466.25 ± 17.52	360.61 ± 14.63
Vinblastine sulfate	24.6 ± 0.65	4.68 ± 0.65

**Table 52:** Anticancer activity of **110**.

Compound	IC_50_ (µM)	
	HepG-2 (Hepatocellular Carcinoma)	MCF-7 (Breast Cancer)
Ligand	>1236.30	>1236.30
**110**	357.90	179.88
Cisplatin	40.76	-
5-fluorouracil	-	215.26

**Table 53:** Anticancer activity of **111a**–**e** (5-FU = 5-fluorouracil).

Cell Line IC_50_ [µM]	Compound					
	111a	111b	111c	111d	111e	5-FU
**Cancer cells**						
**DLD-1**	47.1 ± 4.7	42.7 ± 3.3	65.2 ± 2.5	58.4 ± 5.8	58.8 ± 3.9	50.2 ± 21.0
**HeLa**	5.0 ± 0.4	2.1 ± 0.1	63.9 ± 4.3	115.8 ± 9.2	15.2 ± 1.0	19.2 ± 1.2
**MDA-MB-231**	116.8 ± 14.2	50.0 ± 2.4	48.3 ± 2.5	205.5 ± 25.1	26.0 ± 2.5	22.4 ± 2.5
**HT-29**	198.9 ± 24.3	94.9 ± 9.4	205.1 ± 20.3	198.7 ± 22.5	84.4 ± 8.4	24.2 ± 2.4
**ECC-1**	26.2 ± 2.6	39.5 ± 2.5	362.1 ± 38.1	307.5 ± 34.2	97.6 ± 9.7	30.6 ± 3.5
**DU-145**	104.9 ± 13.5	116.4 ± 14.6	58.3 ± 6.4	160.5 ± 18.0	32.4 ± 3.2	37.3 ± 5.8
**PC-3**	102.0 ± 10.2	65.3 ± 8.5	196.4 ± 22.6	296.8 ± 32.4	79.2 ± 8.5	45.5 ± 4.5
**Normal cells**						
**HEK-239**	68.8 ± 8.7	159.2 ± 19.4	134.7 ± 15.6	196.8 ± 23.5	36.3 ± 3.6	65.3 ± 8.6
**PNT-1A**	76.5 ± 8.5	95.1 ± 11.1	119.5 ± 13.5	125.1 ± 14.8	46.4 ± 4.6	142.3 ± 18.6
**ARPE-19**	119.0 ± 13.5	131.7 ± 15.6	>300	105.2 ± 13.5	84.3 ± 8.4	75.3 ± 7.5

**Table 54:** Antitumor activity of silver(I) complexes with saccharin **112**–**118**.

Compound	IC_50_ [µM]		
	A549 (Lung Carcinoma)	MCF-7 (Breast Adenocarcinoma)	WI-38 (Fibroblast)
**112**	1.18 ± 0.44	1.14 ± 0.19	1.41 ± 0.37
**113**	0.84 ± 0.25	0.82 ± 0.14	0.77 ± 0.25
**114**	1.01 ± 0.12	0.88 ± 0.16	0.74 ± 0.28
**115**	1.79 ± 0.33	3.13 ± 1.55	1.31 ± 0.39
**116**	2.58 ± 0.83	2.32 ± 1.00	9.54 ± 2.40
**117**	9.11 ± 2.32	3.18 ± 1.01	2.61 ± 1.16
**118**	86.4 ± 4.75	5.30 ± 1.61	10.78 ± 2.22
AgNO_3_	21.51 ± 3.63	2.62 ± 1.44	8.14 ± 1.88
Cisplatin	10.92 ± 1.81	4.58 ± 1.25	2.62 ± 1.04

**Table 55:** Antitumor activity of silver(I) complexes **119a**–**e**.

Cell Line IC_50_ [µM]	Compound					
	119a	119b	119c	119d	119e	5-FU
**Cancer cells**						
**DLD-1**	279.3 ± 12.9	31.6 ± 3.2	3.3 ± 0.3	173.3 ± 10.3	110.7 ± 9.0	50.2 ± 21.0
**HeLa**	>300	2.8 ± 0.1	3.4 ± 0.2	139.3 ± 28	9.6 ± 0.6	19.2 ± 1.2
**MDA-MB-231**	96.8 ± 9.6	30.3 ± 3.0	9.8 ± 0.9	>300	148.4 ± 19.5	22.4 ± 2.5
**HT-29**	185.7 ± 16.5	24.9 ± 2.5	10.1 ± 3.1	240.7 ± 28.5	161.6 ± 16.1	24.2 ± 2.4
**ECC-1**	207.0 ± 25.6	29.1 ± 3.0	16.2 ± 1.6	103.6 ± 10.3	135.9 ± 13.5	30.6 ± 3.5
**DU-145**	136.7 ± 16.5	18.6 ± 1.8	4.8 ± 0.4	236.7 ± 28.5	100.3 ± 10.1	37.3 ± 5.8
**PC-3**	612.0 ± 61.2	28.2 ± 3.5	5.1 ± 0.5	136.6 ± 15.8	111.7 ± 13.4	45.5 ± 4.5
**Normal cells**						
**HEK-239**	>300	17.8 ± 1.7	9.9 ± 0.9	5.5 ± 0.6	30.5 ± 4.5	65.3 ± 8.6
**PNT-1A**	209.7 ± 20.9	17.5 ± 17.5	9.2 ± 0.9	139.1 ± 16.5	110.8 ± 11.0	142.3 ± 18.6
**ARPE-19**	>300	22.0 ± 10.2	9.5 ± 0.9	284.8 ± 30.5	109.4 ± 11.0	75.3 ± 7.5

**Table 56:** Antitumor activity of **120**–**124**.

Compound	IC_50_ [µM]	
	A2780/S	A2780/R
**120**	48.2 ± 0.1	54.0 ± 2.4
**121a**	52.7 ± 2.4	44.2 ± 3.2
**121b**	52.4 ± 2.2	40.3 ± 3.7
**122**	8.5 ± 2.4	15.8 ± 0.2
**123**	23.6 ± 1.5	40.8 ± 1.8
**124**	14.9 ± 1.4	14.3 ± 0.9
Cisplatin	2.1 ± 0.3	16.1 ± 0.5

**Table 57:** Anticancer activity of **126** against HepG-2 and HCT-116 cell lines.

Compound	IC_50_ [μg]	
	HepG-2 (Hepatocellular Carcinoma)	HCT-116 (Colon Cancer)
**126**	2.77	3.41
Doxorubicin	0.467	0.471

**Table 58:** Antitumor activity of **127a**–**b** and cisplatin, used as a reference compound.

Compound	GI_50_ [µM]					
	HBL-100	T-47D	HeLa	SW1573	A549	WiDr
**127a**	6.6 ± 1.8	24.0 ± 5.7	3.8 ± 0.8	17.0 ± 2.0	32 ± 2.1	26.0 ± 1.1
**127b**	3.8 ± 0.3	6.5 ± 0.7	3.1 ± 0.5	5.2 ± 0.15	4.7 ± 1.1	9.8 ± 1.2
Cisplatin	1.9 ± 0.16	15 ± 2.3	2.0 ± 0.32	3.0 ± 0.37	1.9 ± 0.64	26 ± 5.3

**Table 59:** Antitumor activity of **128**–**134** against P388 cell line.

Compound	IC_50_ [µM]
	P388 Cell Line
**128a**	0.30
**128b**	>80.13
**129**	52.89
**130**	6.68
**131**	0.22
**132a**	0.33
**132b**	1.58
**133**	4.35
**134**	1.09
Cisplatin	8.15

**Table 60:** Antitumor activity of saccharin complexes with zinc, cadmium and mercury (**135** and **136a**–**b**) after 48h.

Compound	IC_50_ [µM]			
	A549	MCF-7	HT29	MCF10A
**135**	1.74 ± 0.06	3.15 ± 0.10	15.40 ± 0.73	7.94 ± 0.18
**136a**	36.91 ± 0.47	23.01 ± 1.28	36.96 ± 0.75	26.29 ± 1.25
**136b**	80.06 ± 1.35	8.61 ± 0.98	24.27 ± 1.36	42.76 ± 1.43
Cisplatin	5.21 ± 0.18	10.57 ± 0.39	11.25 ± 1.35	13.80 ± 2.36

**Table 61:** IC_50_ values of complexes **137a**–**d.**

Compound	IC_50_ [µM]	
	H-157 (Lung Cancer)	BHK-21 (Hamster Kidney Fibroblasts)
**137a**	1.82 ± 0.11	2.19 ± 0.15
**137b**	2.48 ± 0.13	3.01 ± 1.43
**137c**	3.17 ± 0.28	2.93 ± 0.17
**137d**	1.97 ± 0.17	2.74 ± 0.12
Vincristine	1.08 ± 0.09	1.08 ± 0.09

**Table 62:** Anticancer activity of **79** against MCF-7 and CaCo-2 cell lines.

Compound	IC_50_ (µg/mL)	
	MCF-7	CaCo-2
Sulfaclozine	215.24 ± 0.67	97.6 ± 0.45
**138**	111.91 ± 0.36	198.44 ± 0.25

**Table 63:** Anticancer activity of **145**–**146**.

Compound	IC_50_ [μg]	
	Liver Cancer Cells	Colon Cancer Cells
**145**	0.54	1.54
**146**	0.94	0.67
Doxorubicin	0.4	0.69

**Table 64:** Anticancer activity of **149**–**150**.

Compound	Cell Viability After Treatment (%)	
	MCF-7 (Breast Cancer)	HCEC (Human Corneal Epithelial Cells)
**149**	70.89	26.79
**150**	100	25.72
Ligand	100	22.97

**Table 65:** Antitumor activity of **151**, carbimazole, 6-mercaptopurine and sulfaclozine.

Compound	IC_50_ [µg/mL]
	Caco-2 (Colorectal Adenocarcinoma)
**151**	42.15
Carbimazole	103.477
6-mercaptopurine	98.79
Sulfaclozine	97.6

## Data Availability

Not applicable.
